# Spatiotemporal characterization of aerosols and trace gases over the Yangtze River Delta region, China: impact of trans-boundary pollution and meteorology

**DOI:** 10.1186/s12302-022-00668-2

**Published:** 2022-09-08

**Authors:** Zeeshan Javed, Muhammad Bilal, Zhongfeng Qiu, Guanlin Li, Osama Sandhu, Khalid Mehmood, Yu Wang, Md. Arfan Ali, Cheng Liu, Yuhang Wang, Ruibin Xue, Daolin Du, Xiaojun Zheng

**Affiliations:** 1 Institute of Environment and Ecology, School of the Environment and Safety Engineering, Jiangsu University, Zhenjiang, 212013 China; 2 School of Marine Sciences, Nanjing University of Information Science and Technology, Nanjing, 210044 China; 3 National Agromet Center, Pakistan Meteorological Department, Islamabad, 44000 Pakistan; 4 Key Laboratory of Meteorological Disaster, Ministry of Education [KLME]/Joint International Research Laboratory of Climate and Environment Change [ILCEC]/Collaborative Innovation Center On Forecast and Evaluation of Meteorological Disasters [CIC-FEMD]/CMA Key Laboratory for Aerosol-Cloud-Precipitation, Nanjing University of Information Science and Technology, Nanjing, 210044 China; 5 Department of Precision Machinery and Precision Instrumentation, University of Science and Technology of China, Hefei, 230026 China; 6 Key Laboratory of Environmental Optics & Technology, Anhui Institute of Optics and Fine Mechanics, Hefei Institutes of Physical Science, Chinese Academy of Sciences, Hefei, 230031 China; 7 Center for Excellence in Regional Atmospheric Environment, Institute of Urban Environment, Chinese Academy of Sciences, Xiamen, 361021 China; 8 Key Laboratory of Precision Scientific Instrumentation of Anhui Higher Education Institutes, University of Science and Technology of China, Hefei, 230026 China; 9 School of Earth and Atmospheric Sciences, Georgia Institute of Technology, Atlanta, GA 30332 USA; 10 Shanghai Key Laboratory of Atmospheric Particle Pollution and Prevention [LAP3], Department of Environmental Science and Engineering, Fudan University, Shanghai, 200433 China

**Keywords:** Yangtze River Delta region, Ozone, Particulate matter, China’s clean air action plan, TROPOMI, National Ambient Air Quality Standards

## Abstract

**Background:**

The spatiotemporal variation of observed trace gases (NO_2_, SO_2_, O_3_) and particulate matter (PM_2.5_, PM_10_) were investigated over cities of Yangtze River Delta (YRD) region including Nanjing, Hefei, Shanghai and Hangzhou. Furthermore, the characteristics of different pollution episodes, i.e., haze events (visibility < 7 km, relative humidity < 80%, and PM_2.5_ > 40 µg/m^3^) and complex pollution episodes (PM_2.5_ > 35 µg/m^3^ and O_3_ > 160 µg/m^3^) were studied over the cities of the YRD region. The impact of China clean air action plan on concentration of aerosols and trace gases is examined. The impacts of trans-boundary pollution and different meteorological conditions were also examined.

**Results:**

The highest annual mean concentrations of PM_2.5_, PM_10_, NO_2_ and O_3_ were found for 2019 over all the cities. The annual mean concentrations of PM_2.5_, PM_10_, and NO_2_ showed continuous declines from 2019 to 2021 due to emission control measures and implementation of the Clean Air Action plan over all the cities of the YRD region. The annual mean O_3_ levels showed a decline in 2020 over all the cities of YRD region, which is unprecedented since the beginning of the China’s National environmental monitoring program since 2013. However, a slight increase in annual O_3_ was observed in 2021. The highest overall means of PM_2.5_, PM_10_, SO_2_, and NO_2_ were observed over Hefei, whereas the highest O_3_ levels were found in Nanjing. Despite the strict control measures, PM_2.5_ and PM_10_ concentrations exceeded the Grade-1 National Ambient Air Quality Standards (NAAQS) and WHO (World Health Organization) guidelines over all the cities of the YRD region. The number of haze days was higher in Hefei and Nanjing, whereas the complex pollution episodes or concurrent occurrence of O_3_ and PM_2.5_ pollution days were higher in Hangzhou and Shanghai.

The in situ data for SO_2_ and NO_2_ showed strong correlation with Tropospheric Monitoring Instrument (TROPOMI) satellite data.

**Conclusions:**

Despite the observed reductions in primary pollutants concentrations, the secondary pollutants formation is still a concern for major metropolises. The increase in temperature and lower relative humidity favors the accumulation of O_3_, while low temperature, low wind speeds and lower relative humidity favor the accumulation of primary pollutants. This study depicts different air pollution problems for different cities inside a region. Therefore, there is a dire need to continuous monitoring and analysis of air quality parameters and design city-specific policies and action plans to effectively deal with the metropolitan pollution.

**Supplementary Information:**

The online version contains supplementary material available at 10.1186/s12302-022-00668-2.

## Background

Rapid urbanization and industrialization, deteriorating the air quality of most Chinese cities have resulted in adverse impacts on public health [1–3]. According to the World Health Organization [WHO] standards, only 1% of Chinese megacities meet the safe city criteria in terms of air quality [4]. Atmospheric aerosols unswervingly impact the earth’s radiation budget, ecological environment, human health, and climate [5]. Atmospheric pollution is caused by higher concentrations of various trace gas species including the oxides of nitrogen (NO_x_) and sulfur (SO_x_), tropospheric ozone (O_3_), volatile organic compounds (VOCs), and airborne particulate matter [PM], which pose serious threats to human health [6–8]. The primary emissions from anthropogenic sources are the trace gases such as NO_2_, SO_2_, and carbon monoxide (CO) [9]. Industrial emissions, fossil fuel combustion, and biofuels act as the major sources of SO_2_ and CO [10]. Both CO and NO_x_ act as the main precursors of O_3_, whereas NO_X_ and SO_2_ are significant towards the production of secondary inorganic aerosols [11]. The pollution sources have a significant impact on aerosol properties and are driven by atmospheric oxidation capacity, the intensity of emissions, and meteorological conditions. The role of meteorological parameters on different pollution episodes have been documented in previously reported studies [11]. Despite ample research on the effects of meteorological parameters on atmospheric pollution, significant variation in the intensity and extent of these impacts exists for different seasons and across different regions.

China has established more than 1500 stations for air quality monitoring since 2013, with a prime focus on monitoring different trace gases and particulate matter, i.e., PM_2.5_, PM_10_, NO_2_, SO_2_, O_3_, and CO [10–13]. The measurements taken at these monitoring stations have been used to analyze the characteristics of different air pollution episodes in various regions of China including the Yangtze River Delta (YRD) region [10–12, 14].

The YRD region is located adjacent to the North China Plain (NCP) and experiences complicated environmental pollution issues originating from dust plumes, biomass burning, photochemical reactions, and coal combustion [15]. The occurrence of severe haze episodes in the YRD region is one of these environmental pollution issues [16–18]. Haze is a condition that obstructs visual range and is defined as the weather phenomenon that leads to a reduction in atmospheric visibility caused by the accumulation of suspended solid or liquid fine particles [19–21]. The other major pollution issue in the YRD region is a phenomenon in which high O_3_ and high PM_2.5_ coincided beyond the National Ambient Air Quality Standards (NAAQS) [14, 22, 23]. This condition is termed as a complex pollution in this study hereafter, where the daily averaged PM_2.5_ concentration exceeds 35 μg/m^3^, and the daily averaged O_3_ concentration exceeds 160 μg/m^3^ on the same day.

The effects of cross-boundary air pollution and climatic factors on air quality are reported in different cities of the YRD region [24–27]. Though these investigations were either limited to specific particulate matter, trace gases, time intervals, or certain regions of YRD, the variation among various cities of YRD and their influential factors are not still clear. Thus, it is imperative to study the recent spatiotemporal distribution and characteristics of the criteria pollutants at different geographical locations within YRD. This is the first comprehensive study analyzing different air pollution episodes across YRD regions using recent datasets from National environmental monitoring centers. The study broadly characterizes the current air quality status, over the four most polluted and populous cities of YRD during 2018–2021. This research (a) examines the recent long-term temporal variation of trace gases and particulate matter (b) impact of different meteorological parameters on pollutants (c) identifies different pollutant sources using PSCF (Potential Source Contribution Function) with HYSPLIT (Hybrid Single-Particle Lagrangian Integrated Trajectory) back trajectory analysis (d) in situ measurements of SO_2_ and NO_2_ are compared with TROPOMI satellite data. Further, haze and complex pollution episodes over the study region were also analyzed. The outcomes of this up-to-date study could provide a wide-ranging logical and scientific basis for decision-making while designing effective air pollution control policies for metropolises.

## Methods

### Description of the study area

**Fig. 1:**
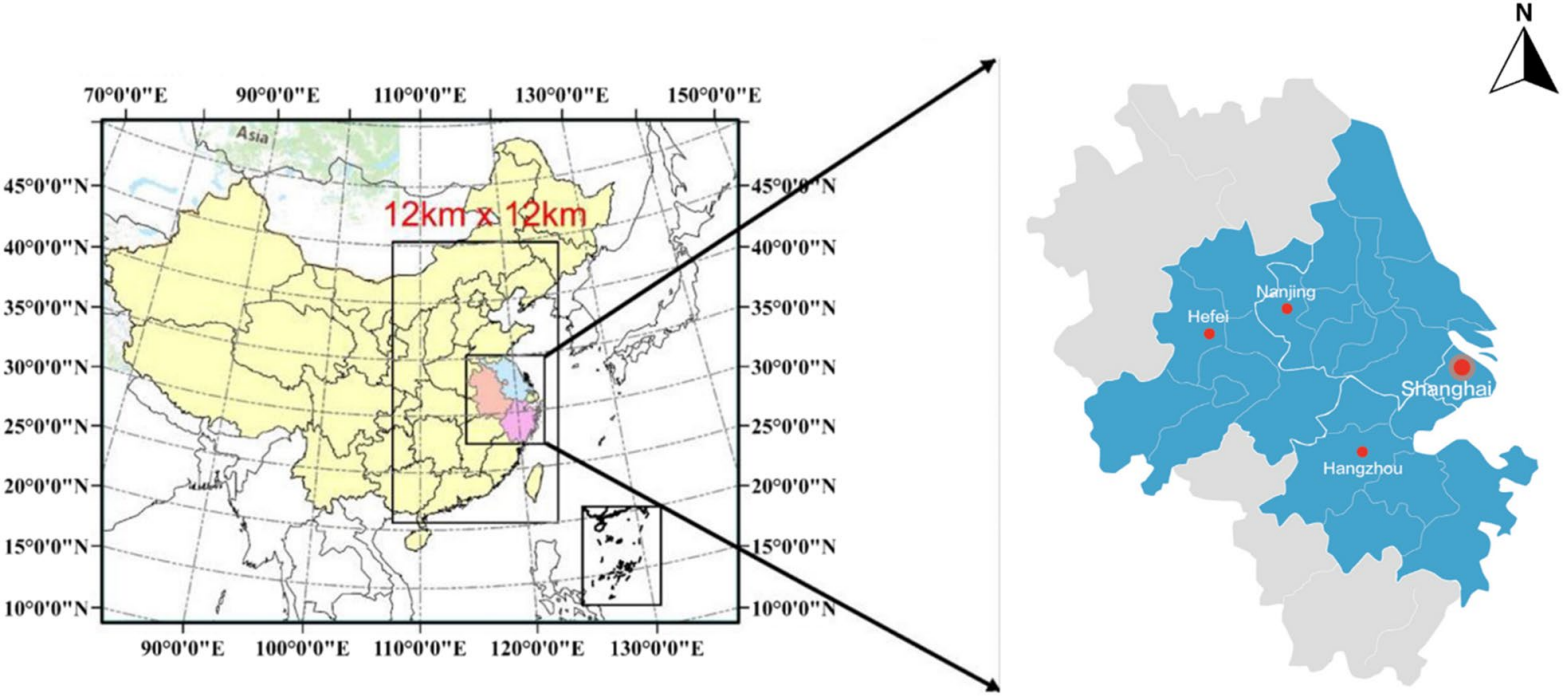
The location of four megacities in YRD region of China

Anhui, Jiangsu, Shanghai, and Zhejiang are the major provinces that mainly cover the eastern side of the Yangtze plain. Fertile plains make this area characteristically unique and deem it suitable for a variety of food as well as cash crops. According to statistics, the gross domestic product (GDP) exceeded over USD 2.2 trillion for the YRD region as of 2018 (China Statistics: http://data.stats.gov.cn/english, accessed on 26 December 2021). The total area covered by the YRD region sums up to be 210,700 km^2^ and supports a huge population numbered about 0.23 billion in 2018 [28]. The vulnerability of the region to excessive atmospheric pollution comes from the high industrial growth, economic activity, and ever-increasing population which enhances the anthropogenic footprint on the environment. The four most important cities of the YRD region have been observed in this study, including Hangzhou, Hefei, Nanjing, and Shanghai. All of these have a very high population density and are the hubs for industrial and economic activities which make them prone to extreme pollution episodes. Figure 1 shows the location of these above-mentioned megacities in the YRD region.

### Data sets

#### In situ measurements and meteorological parameters

In situ measurements were attained via a National online database for air quality analysis and monitoring platform for NO_2_, PM_2.5_, PM_10_, SO_2_, and MDA8 O_3_ (Maximum Daily 8-hour Average Ozone). The data are available online and the monitoring stations are managed by China National Environmental Monitoring Center. The instruments deployed at these monitoring centers accord to the China Environmental Protection Standard HJ 664-2013. National ambient air quality standards (GB 3095e2012; China) were used to check the validity of the data, as reported in the previous study [12]. The hourly mean data of 10 different monitoring stations over each city (Additional file 1: Table S1) were used from 2018 to 2021. The city-wide day-to-day mean level of pollutants was estimated by taking an average of all the monitoring stations over a particular city as reported in previous studies [29]. The quality control approaches were applied to filter the problematic data as described in previous studies [30].

Meteorological parameters including temperature, visibility, relative humidity and wind speed were taken into account for the aforementioned time frame. Automatic weather stations installed at respective airports of each city were used to obtain meteorological data.

#### TROPOMI

TROPOMI level-2 NO_2_ and SO_2_ product published by Royal Netherlands Meteorological Institute (KNMI) has been used for the current study. Differential optical absorption spectroscopy (DOAS) algorithm is used to retrieve tropospheric VCDs following three major steps: 1) retrieval of slant column densities (SCDs), 2) differentiating stratospheric SCDs from tropospheric SCDs, and 3) converting SCDs to VCDs using air mass factor (AMF). Owing to the error linked to the calculation of AMF, an obvious underestimation of TROPOMI tropospheric NO_2_ and SO_2_ has been reported in recent studies, especially in polluted regions. In order to ensure reliability, the data with quality flag exceeding 0.75 were used for this study.

### Methodology

#### Categorization of different pollution episodes

##### Haze pollution

To investigate the impact of haze days on PM_2.5_, PM_10_, NO_2_, SO_2_, and O_3_ over the study region, the study period has been divided into the haze and non-haze days. Haze days were defined with visibility < 7.5 km, RH < 80%, and PM_2.5_ > 40 µg/m^3^, while non-haze days were defined with visibility > 7.5 km, RH < 80%, and PM_2.5_ < 40 µg/m^3^ non-haze. A similar categorization of Haze and non-haze days is reported in different studies [19, 20, 31]. The aforementioned categorization for haze and non-haze days has been used for the current study over main cities of the YRD region including Hangzhou, Hefei, Nanjing, and Shanghai.

##### Complex pollution

The complex pollution days refer to the pollution conditions where the daily averaged PM_2.5_ concentration exceeds 35 μg/m^3^, and the daily averaged O_3_ concentration exceeds 160 μg/m^3^ on the same day. This categorization is used in this study over cities of the YRD region including Hangzhou, Hefei, Nanjing, and Shanghai.

#### Principal component analysis

Possible pollutant sources can be explored by employing principal component analysis (PCA) along with the study of the correlations among the pollutant concentrations. PCA was employed using SPSS v17.0 for five air pollutants with daily mean data points for each variable. The numbers of principal components (PCs) are determined by the variability in the data and frequency of inspected elements. PCA exploits the factor loading of each variable, which is why it was used with Varimax rotation [32] as reported in the earlier study [33].

#### Potential source contribution function

The origin of air masses was determined by performing backward trajectory analysis by employing the Hybrid Single-Particle Lagrangian Integrated Trajectory (HYSPLIT) model established by National Oceanic and Atmospheric Administration (NOAA) [34]. A comprehensive understanding of the transport of air masses, their dispersion, and chemical conversion is offered by this model [35] which also highlights the probable causes of aerosol pollutants that impact the air quality. The integration of HYSPLIT backward trajectory with PSCF analysis and daily SO_2_, NO_2_, O_3_, PM_2.5_, and PM_10_ measurements over a grid of 0.5*0.5 degrees was carried out to find the source strength of different geographical locations. The trajectory was calculated (72 h for every 6 h) at a height of 500 m above the ground level (AGL) employing meteorological data from the GDAS (Global Data Assimilation System) on 1◦ × 1◦ spatial resolution (accessed from: ftp://arlftp.arlhq.noaa.gov/pub/archives/gdas1) for each season from January 2018 to December 2021. Weighting functions were performed to lessen the uncertainty of PSCF and denoted as WPSCF [36, 37].

## Results and discussion

### Temporal variation

**Fig. 2:**
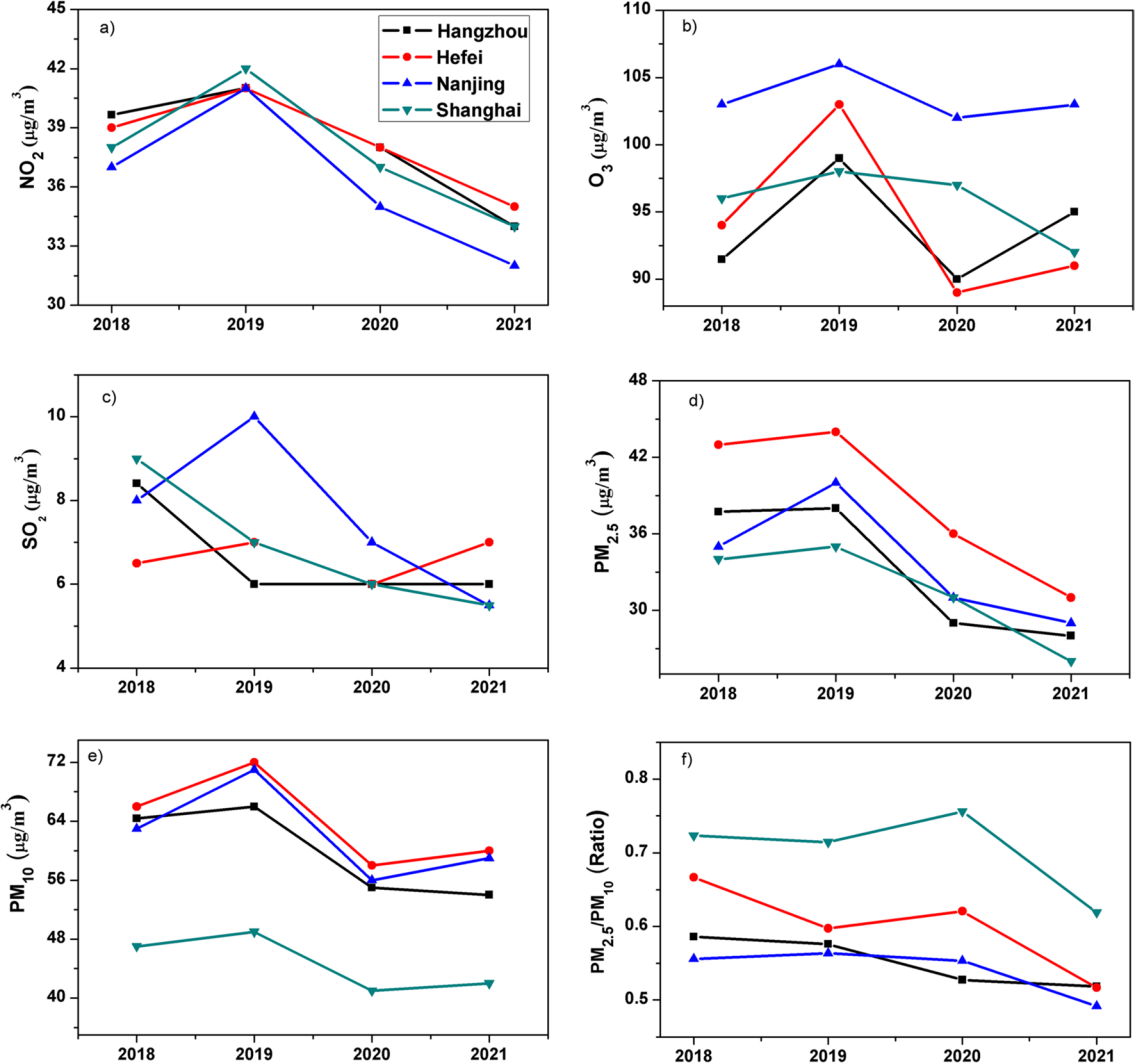
The inter-annual variation of **a** NO_2_, **b** O_3_, **c** SO_2_, **d** PM_2.5_, **e** PM_10_ and **f** PM_2.5_/PM_10_ in Hangzhou, Nanjing, Hefei and Shanghai during 2018–2021

The time-series for yearly mean and inter-annual temporal trends of NO_2_, MDA8 O_3_, SO_2_, PM_2.5_, PM_10_, and PM_2.5_/PM_10_ from 2018–2021 are shown in Fig. 2. The basic statistics (annual mean, maxima, minima, standard deviation, and median) of the daily mean (2018-2021) NO_2_, MDA8 O_3_, SO_2_, PM_2.5_, and PM_10_ are shown in Additional file 1: Tables S2–S5 for Hefei, Nanjing, Shanghai, and Hangzhou. The main sources for NO_2_ production are generally vehicular emissions, biomass burning, fossil fuel combustion, and industrial emissions [36, 38]. The highest annual mean NO_2_ concentration (42 µg/m^3^) over Shanghai was observed in 2019, whereas 19% decline was observed from 2019 to 2021. Similar temporal variations were observed over other cities, i.e., the highest annual mean NO_2_ concentration was observed in 2019 and decreased by 17%, 21%, and 14% over Hangzhou, Nanjing, and Hefei, respectively, from 2019 to 2021. Only in 2019, the mean NO_2_ concentrations exceed the National Ambient Air Quality Standards (NAAQS< 40 µg/m^3^) and World Health organization Standards (WHO<40µg/m^3^) over all the cities. However, for the rest of the years, all the cities meet the NAAQS and WHO air quality standards for NO_2_. The decreasing trend in NO_2_ can be accredited to the efficacy of emission control measures and implementation of the Clean Air Action plan of China. The regulation of NO_x_ emissions from power plants played an important role in the reduction of NO_2_ [39]. Overall, these results show that Hefei (38.4 µg/m^3^) is the most polluted city of the YRD region compared to the other cities, however, all the cities meet the NAAQS and WHO air quality standards based on annual mean NO_2_ concentrations from 2018 to 2021.

SO_2_ is mainly produced from fossil fuel burning for domestic heating, power plants emissions, oil refineries, and metal smelters [40]. Across the four cities, the highest annual mean SO_2_ concentration (10 µg/m^3^) was observed in Nanjing during 2019. The annual mean concentration of SO_2_ in Nanjing decreased by 45% from 2019 to 2021. The temporal variation of SO_2_ over different cities of the YRD region showed contrasting results. The maxima for Hangzhou and Shanghai (8.5 and 9 µg/m^3^) were observed in 2018, whereas the decline was 28% and 38%, respectively, from 2018 to 2021. The annual mean SO_2_ concentration from 2018 to 2021 in Hefei on a whole ranged from 6 to 7 (µg/m^3^). The highest value 7 µg/m^3^ was observed in both 2018 and 2021. The SO_2_ concentration in all the cities was below the NAAQS (20 µg/m^3^) and WHO (20 µg/m^3^) guidelines. Additional file 1: Table S6 shows the China NAAQS and WHO guidelines for different trace gases and particulate matter. Previous studies also reported a decline in mean SO_2_ concentration over different regions of China [12]. Concerning, the most polluted city during 2018–2021 in terms of SO_2_ pollution was Nanjing with an overall mean concentration of 7.6 µg/m^3^. The reduction in SO_2_ levels over the YRD region can be linked to the strong and effective measures taken to reduce the SO_2_ level. The previous study reported a decrease in industrial SO_2_ emissions following the widespread installation of fuel gas desulfurization (FGD) devices in China [41].

Temporal variations of PM_2.5_ concentrations show a similar pattern as NO_2_, i.e., the highest and lowest annual mean concentrations in 2019 and 2021, respectively. The annual mean PM_2.5_ concentration decreased from 2019 to 2021 by 29%, 27%, 26%, and 25% over Hefei, Nanjing, Hangzhou, and Shanghai, respectively. The lowest mean yearly concentration of PM_2.5_ (26 µg/m^3^) was observed in Shanghai. The PM_2.5_ concentration was substantially reduced over all the YRD cities. Similar findings were reported in earlier studies [22, 42]. The air pollution prevention and control action plan launched by the Chinese government in 2013 has helped in significant PM_2.5_ emission abatements. However, the yearly mean concentration of PM_2.5_ still exceeds the Grade 1 annual NAAQS (15 µg/m^3^) and WHO (15 µg/m^3^) air quality guidelines over all the cities.

The yearly mean PM_10_ concentration for all the cities varies abruptly, i.e., PM_10_ concentration increased in 2019 compared to 2018, decreased in 2020, and then again increased in 2021. However, the lowest annual mean PM_10_ was noted in 2020. The highest annual mean PM_10_ (72 µg/m^3^) was observed in 2019 over Hefei compared to the other cities. The annual mean PM_10_ levels were beyond the grade 1 NAAQS (40 µg/m^3^) and WHO (15 µg/m^3^) guidelines over all the cities of YRD. This indicates that all the cities are facing severe air pollution problems in terms of PM_2.5_ and PM_10_.

The highest annual mean concentration of MDA8 O_3_ (106 µg/m^3^) was observed in 2019 over Nanjing. There has been an unprecedented reduction in average O_3_ concentrations during 2020 over all the cities of YRD, while the previously reported studies depicted a continuous increase of approximately 5% in annual mean concentration from 2013 to 2019 over different parts of China [22, 43]. However, a recent study also reported an exceptional reduction of approximately 6% in summer time MDA8 O_3_ during 2020 compared to 2019 over different Chinese cities [44]. This unprecedented decline in MDA8 O_3_ during 2020 can be linked to the fact that a recent study reported a shift in ozone sensitivity from the VOC (volatile organic compounds) limited regime to the transitional regime over eastern China [45] indicating that concurrent control of both NO_x_ and VOCs would benefit in ozone reductions. Therefore, concurrent reduction of both NO_x_ and VOCs during 2020 due to ongoing mitigation plan and new stringent control measures for 2020 introduced by the Chinese Ministry of Environment and Ecology in 2020 [46] may be helped in the reduction of ozone level. However, the MDA8 O_3_ levels again showed a slight increase during 2021 compared to 2020 over Hefei, Nanjing, and Hangzhou; whereas, Shanghai showed a continuous reduction in MDA8 O_3_ concentration. Therefore, it is still not clear which specific measures have helped in the reduction of O_3_ and requires further studies over different parts of China.

The daily average concentrations of PM_10_ and PM_2.5_ from 2018 to 2021 were used to analyze the ratio of PM_2.5_ to PM_10_ (PM_2.5_/PM_10_) to assess PM pollution over the studied cities (Shanghai, Nanjing, Hefei, and Hangzhou). Domestic heating and lower temperatures contributed to high PM_2.5_/PM_10_ values and their rate of change during winters in general. Due to high penetration rates and enhanced diffusion, smaller particles are more harmful compared to large particles. Therefore, it is imperative to reduce the proportion of PM_2.5_ in PM_10_ by applying control strategies especially focusing on the reduction by vehicular discharge and industrial emissions which mainly contribute to PM_2.5_. Domestic heating, traffic, spatial location, and meteorology should be simultaneously considered while conducting the measures to reduce PM pollution. The larger PM_2.5_/PM_10_ are observed in Shanghai and Hefei. This is an indication that fine particulate matter from anthropogenic sources mainly influences the air quality of Hefei and Shanghai.

It is worth discussing here that COVID-19 lockdown also played a significant part in the decline of the annual mean concentration of trace gases and particulate matter during 2020. Several studies reported the impact of COVID-19 lockdown on the concentration of trace gases and particulate matter over the YRD region [47–49]. Therefore, the annual time-series of particulate matter and other trace gases were also generated excluding the COVID-19 lockdown period (i.e., 24th of January–31st of March) from each year (Additional file 1: Figure S1). The results depict that the fewer decline in annual mean concentration during 2020 is observed in time-series excluding lockdown period as compared to time-series including the COVID-19 lockdown period. Additional file 1: Table S7 shows the change in percentage of annual mean concentrations from 2019 to 2020 for whole year and excluding COVID-19 lockdown period. The results indicate that for both whole year and excluding COVID-19 lockdown period the change in percentage of trace gases and particulate matter is negative, i.e., decline in concentration is observed. However, the results indicate that decline in concentration of NO_2,_ SO_2,_ PM_2.5_ and PM_10_ is relatively less when COVID-19 lockdown period is excluded. This shows the importance of COVID-19 lockdown which resulted in improvement of air quality. Contrasting results were noticed for O_3_ which showed higher decline in concentration when COVID-19 lockdown period is excluded. The previous study over Nanjing also showed increase in concentration of O_3_ during COVID-19 lockdown period [47].

### Haze pollution

**Fig. 3:**
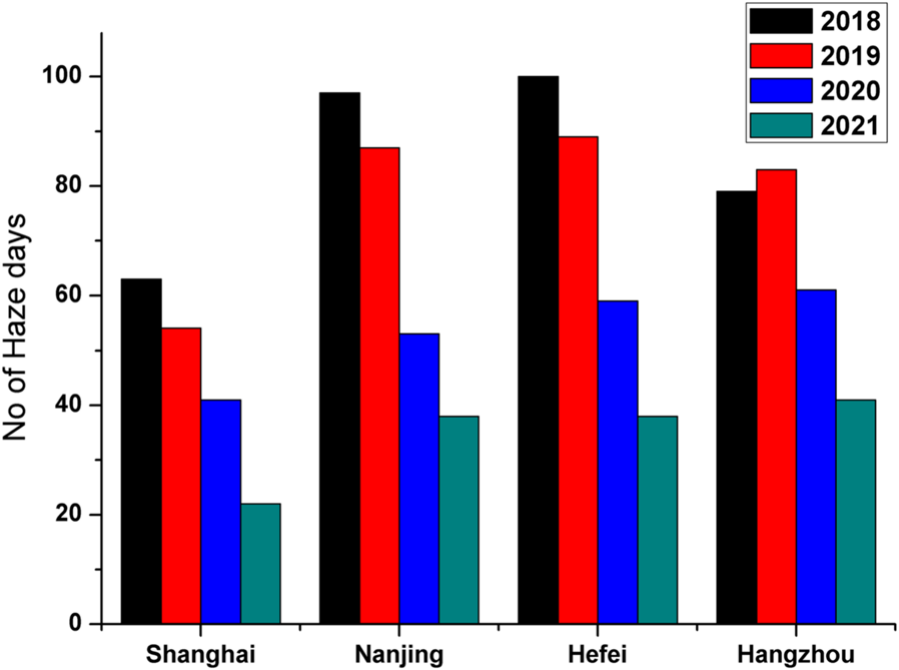
The number of haze days over different cities of YRD region

Haze formation is one of the phenomena that is largely impacted by the meteorological conditions of the locality. This haze impacts pollutants distribution, residence-time and photochemical reactions, thereby altering the atmospheric composition and impacting air quality [50, 51]. The study period is categorized into haze and non-haze days according to the categorization described above in the methodology section. Fig. 3 shows the number of haze days for all the four cities studied during the four consecutive years. Evidently, Hefei and Nanjing have the highest number of haze days, while Shanghai and Hangzhou have considerably fewer haze days compared to other cities. However, the general trend shows an average drop in haze days occurrence from 2018 to 2021.

Further, the impact of the haze condition on the concentration of trace gases and particulate matter is also examined. Table 1 depicts the percent change in mean concentrations of NO_2_, SO_2_, O_3_, PM_2.5_, and PM_10_ during haze days.

**Table 1:** Comparison of criteria pollutants during haze and non-haze days (2018–2021) over the YRD region cities

Cities	Pollutant		Percent change
	Mean PM_2.5_ concentrations [µg/m^3^]		
	Haze days[H]	Non-haze days[N]	[H-N]/N*100
Shanghai	77	28	175%
Nanjing	60	27	122%
Hefei	45	26	73%
Hangzhou	48	28	72%
Mean SO_2_ concentrations [µg/m^3^]			
Shanghai	10	07	42%
Nanjing	9.7	8.5	15%
Hefei	7	6.25	12%
Hangzhou	7.77	6.75	15%
Mean NO_2_ concentrations [µg/m^3^]			
Shanghai	56	35	60%
Nanjing	48	33.5	45%
Hefei	45	35	28%
Hangzhou	47	32	46%
Mean PM_10_ concentrations [µg/m^3^]			
Nanjing	95	60	58%
Hefei	75	57	31%
Hangzhou	80	52.5	53%
Mean O_3_ concentrations [µg/m^3^]			
Shanghai	94	103	− 9%
Nanjing	93	123	− 32%
Hefei	90	113	− 25%
Hangzhou	85	111	− 30%

The concentration of all the particulate matter and trace gases except O_3_ is higher during haze days. This increase in concentration is mainly because meteorological conditions like low wind speed and high relative humidity were noted during haze days, which were favorable to the accumulation of air pollutants [52]. However, O_3_ concentrations tend to be higher during non-haze days compared to haze days over all the cities. During haze days, the light intensity is low and the conditions are less likely to favor photochemical reaction, hence the rate of O_3_ formation is low compared to non-haze sky conditions when ample sunlight is available [53]. The highest change is observed in the concentration of PM_2.5_.

### Complex pollution episodes

**Fig. 4:**
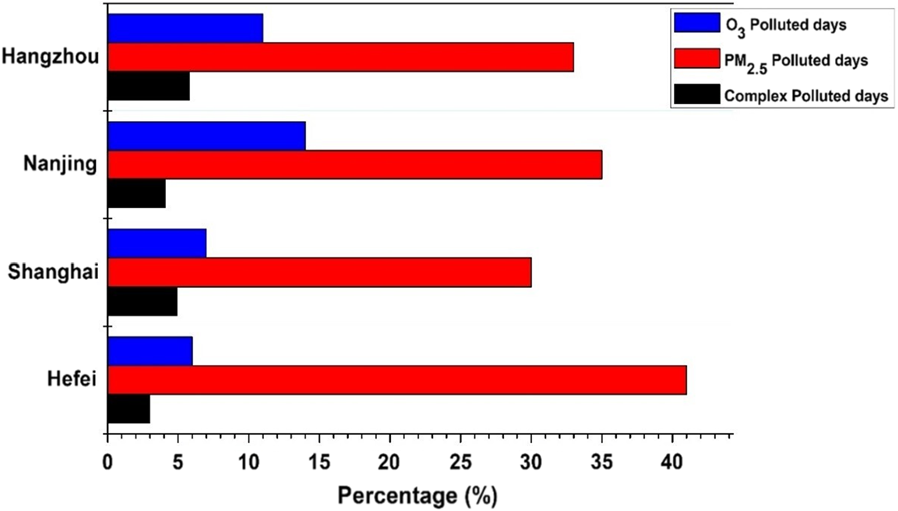
The percentage of occurrence of O_3_ polluted days, PM_2.5_ polluted days and complex polluted days over different cities of YRD from 2018 to 2021

The higher concentrations of O_3_ and PM_2.5_ have adversative effects on community health and the environment [14, 54]. The complex pollution episode is basically the co-occurrence of both PM_2.5_ and MDA8 O_3_ at higher levels. The complex pollution episodes are identified in the study period according to the categorization described in the methodology section. Fig. 4 shows the percentage of occurrence of O_3_ polluted days (O_3_ > 160 μg/m^3^), PM_2.5_ polluted days (PM_2.5_ > 35 μg/m^3^) and complex polluted days (O_3_ > 160 μg/m^3^ and PM_2.5_ > 35 μg/m^3^) over Shanghai, Nanjing, Hefei and Hangzhou.

**Fig. 5:**
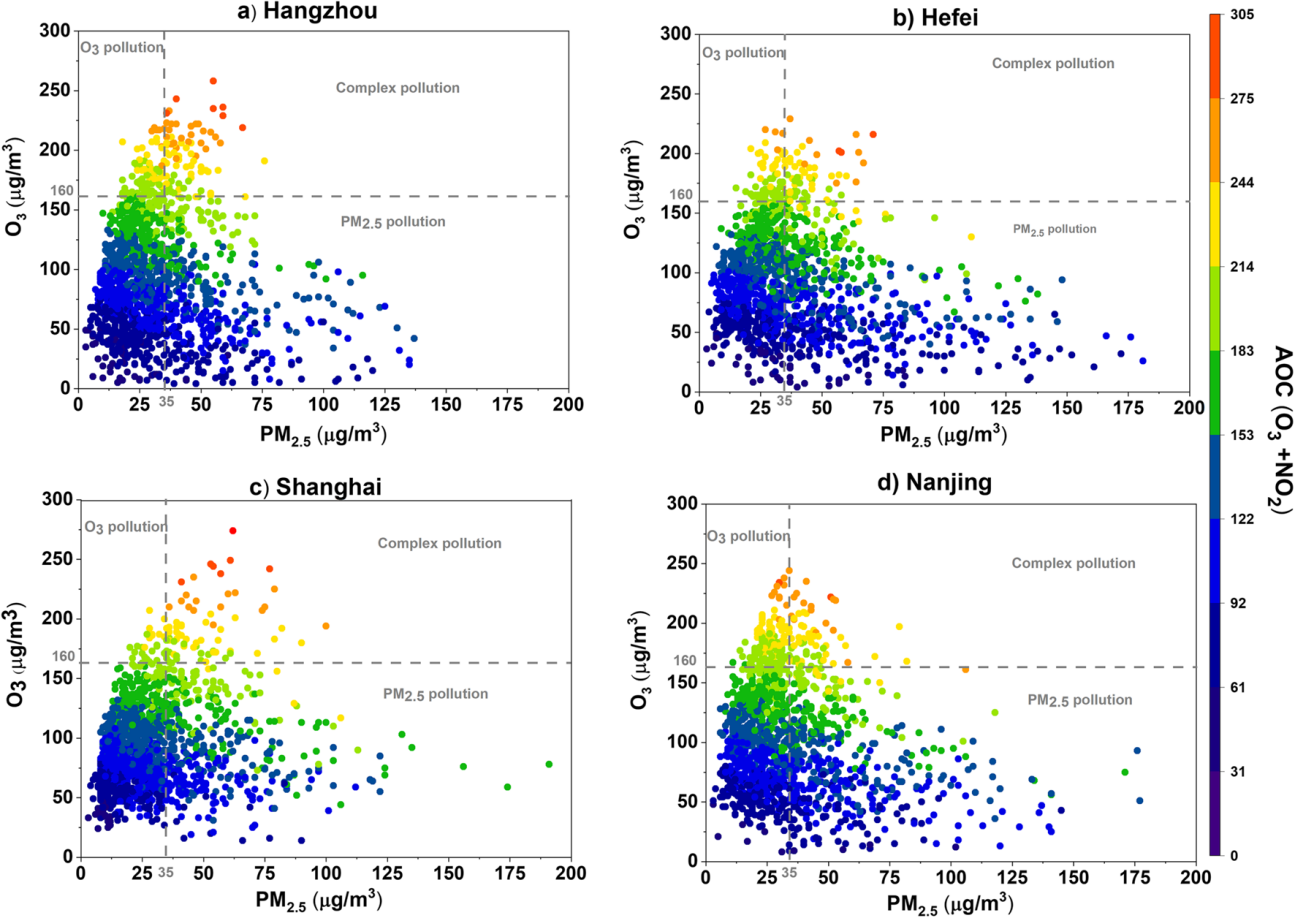
The scatter plot of daily mean PM_2.5_ and MDA8-O_3_ with atmospheric oxidation capacity (O_x_) is indicated by color. The dashed lines depict the NAAQS grade-II standards for O_3_ and PM_2.5_

The results show that the highest number of PM_2.5_ polluted days is observed in Hefei (41%), whereas the O_3_ and complex polluted days are lowest in Hefei. The highest percentage of O_3_ polluted days (14%) occurred in Nanjing compared to other cities. However, the highest percentage of complex polluted days occurred in Hangzhou (5.8%) and Shanghai (5%), compared to Hefei (3%) and Nanjing (4%). The primary pollutants concentration (NO_2_ and SO_2_) is lower in Hangzhou and Shanghai as depicted in the previous section but the percentage of complex pollution days is higher. This is an indication that despite a reduction of primary pollutants in the main cities of China, the formation of the secondary pollutant is still a concern. During the complex pollution, days mean O_X_ (O_3_+NO_2_) is compared for different cities, which is an indicator of atmospheric oxidation capacity. The mean O_X_ levels (atmospheric oxidation capacity) during complex pollution episodes indicate that Hangzhou and Shanghai have higher oxidation capacity during complex pollution days compared to Nanjing and Hefei as shown in scatter plot of daily mean PM_2.5_ and MDA8-O_3_ with atmospheric oxidation capacity (O_x_) in Fig. 5. It is pertinent to mention that PM_10_ and MDA8- O_3_ are also closely linked but this study mainly focused on complex pollution episodes, i.e., the co-occurrence of both PM_2.5_ and MDA8 O_3_ at higher levels, therefore, the scatter plots of daily mean PM_2.5_ and MDA8-O_3_ are presented in this study. The higher oxidation capacity can enhance the rate of secondary pollution formation. Similar results were reported in previous studies conducted in China [52].

### Influence of meteorology

**Fig. 6:**
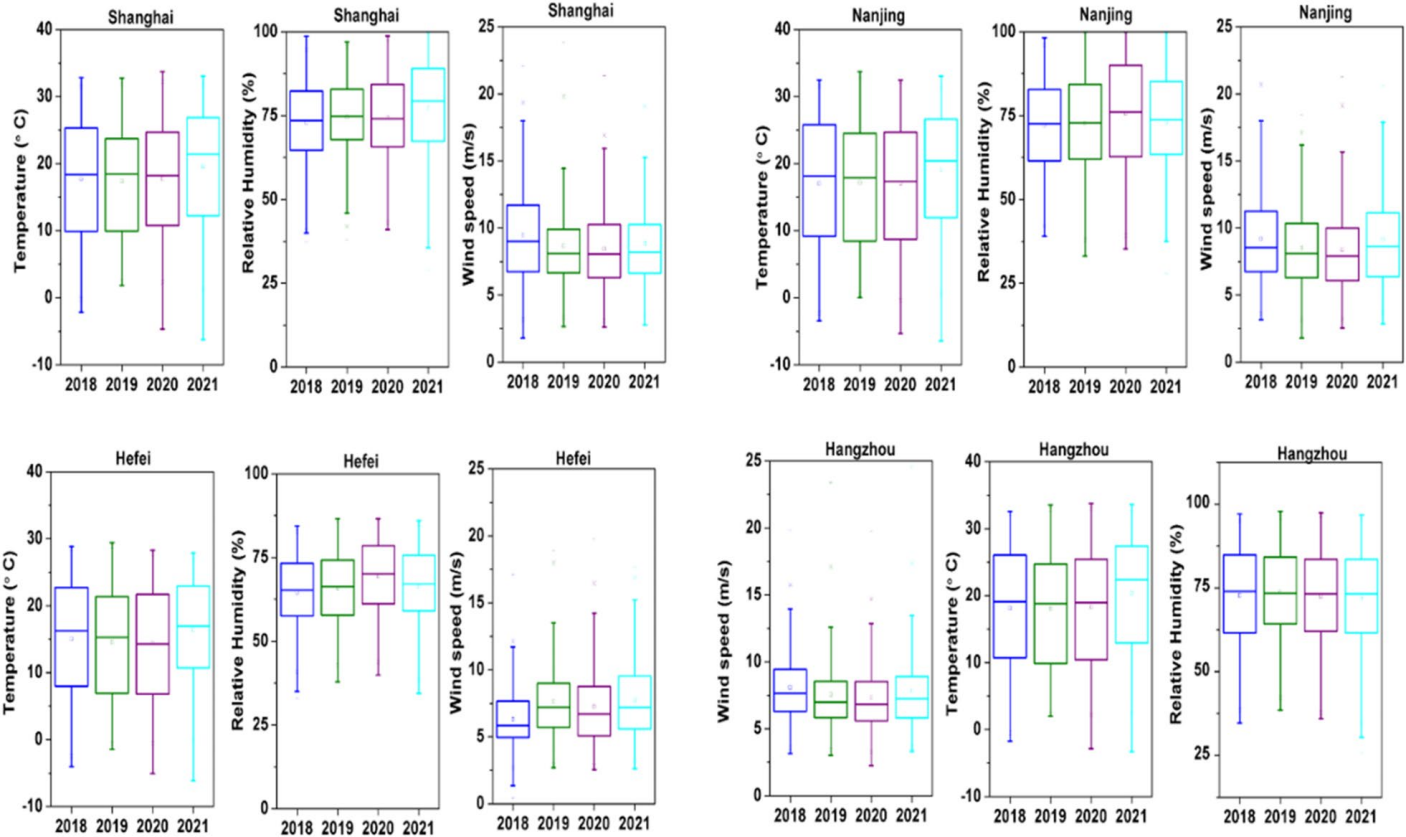
Box plot of different meteorological parameters from 2018 to 2021 over different cities of YRD

Meteorological factors play a key role in trace gas and aerosol distribution in the atmosphere and substantial pieces of evidence can be found in the literature pertaining to the impact of meteorology on local air quality [55]. Therefore, inter-annual variation in different meteorological parameters like temperature, relative humidity, and wind speed is observed. Fig. 6 shows box plots of meteorological parameters from 2018 to 2021. The results show that there is no significant change in inter-annual variation of temperature, relative humidity, and wind speed. The meteorological conditions are almost identical from 2018 to 2021 over YRD region cities. This provided limited assurance that the air quality trends are not partially influenced by year-to-year meteorological differences. Therefore, inter-annual variations in trace gases and particulate matter are possibly due to changes in emission sources and strict measures taken by the Chinese Government.

However, the meteorological conditions at the sub-seasonal scale of a locality are considered to be a major driving force towards the distribution of pollutants in terms of residence-time and chemical behavior in the lower atmosphere. Therefore, the pairwise correlation analysis at a seasonal scale was used to understand the relationship among meteorological factors and five criteria pollutants (NO_2_, SO_2_, O_3_, PM_2.5_, and PM_10_). The correlation coefficients between pollutants and meteorological factors are depicted in Table 2. Strong negative correlation of wind speed was observed with NO_2_, especially during summer and winters, compared to other parameters. The wind speed is negatively correlated with primary pollutants because lower wind speeds result in the accumulation of different primary pollutants [55]. The secondary pollutant O_3_ is also weakly and negatively correlated with wind speed except in the autumn season. The trend is similar over all the cities.

**Table 2:** Pearson correlation coefficient between air pollutants and meteorological factors (2018–2021)

Pollutant	Temperature[°C]				Relative humidity [%]				Wind speed [m/s]			
Shanghai	Spring	Summer	Winter	Autumn	Spring	Summer	Winter	Autumn	Spring	Summer	Winter	Autumn
PM_2.5_	0.110	− 0.110	− 0.084	− 0.225	− 0.003	− 0.159	− 0.115	0.005	− 0.242	− 0.450	− 0.218	− 0.264
PM_10_	0.171	0.299	− 0.191	− 0.146	− 0.403	− 0.511	− 0.415	− 0.260	− 0.125	− 0.220	− 0.174	− 0.258
NO_2_	− 0.013	− 0.224	0.031	− 0.346	− 0.063	0.097	− 0.086	0.083	− 0.439	− 0.675	− 0.429	− 0.575
SO_2_	0.251	0.116	− 0.327	− 0.032	− 0.307	− 0.548	− 0.458	− 0.313	− 0.107	− 0.042	− 0.145	− 0.211
O_3_	0.551	0.162	0.226	0.365	− 0.550	− 0.525	− 0.173	− 0.425	− 0.005	− 0.283	0.069	− 0.164
Hefei												
PM_2.5_	− 0.138	0.071	0.02	− 0.211	0.059	− 0.330	0.13	− 0.047	− 0.068	− 0.311	− 0.25	− 0.224
PM_10_	0.138	0.336	0.09	− 0.028	− 0.472	− 0.635	− 0.27	− 0.427	− 0.051	− 0.266	− 0.26	− 0.302
NO_2_	0.037	− 0.070	0.02	− 0.260	− 0.382	− 0.245	− 0.23	− 0.333	− 0.298	− 0.524	− 0.38	− 0.547
SO_2_	0.117	0.248	0.04	− 0.049	− 0.512	− 0.475	− 0.59	− 0.464	− 0.070	− 0.059	− 0.24	− 0.248
O_3_	0.652	0.266	0.19	0.692	− 0.590	− 0.744	− 0.43	− 0.463	− 0.177	− 0.224	0.19	− 0.176
Nanjing												
PM_2.5_	0.05	− 0.006	0.002	− 0.23	0.02	− 0.20	0.05	0.01	− 0.19	− 0.20	− 0.25	− 0.12
PM_10_	0.43	0.15	0.016	− 0.19	− 0.32	− 0.46	− 0.17	− 0.34	− 0.21	− 0.15	− 0.23	0.02
NO_2_	0.19	− 0.03	0.071	− 0.28	− 0.33	− 0.11	− 0.13	− 0.31	− 0.36	− 0.45	− 0.40	− 0.33
SO_2_	0.0016	0.25	− 0.08	0.12	− 0.40	− 0.60	− 0.41	− 0.45	− 0.32	− 0.05	− 0.22	− 0.20
O_3_	0.26	0.26	0.17	0.67	− 0.61	− 0.70	− 0.43	− 0.42	− 0.08	− 0.09	0.17	− 0.05
Hangzhou												
PM_2.5_	0.056	0.003	− 0.015	− 0.022	− 0.110	− 0.154	− 0.093	− 0.097	− 0.165	− 0.349	− 0.246	− 0.39
PM_10_	0.108	0.112	0.078	− 0.048	− 0.375	− 0.295	− 0.242	− 0.290	− 0.134	− 0.284	− 0.228	− 0.40
NO_2_	− 0.013	− 0.382	0.076	− 0.269	− 0.091	0.252	− 0.021	− 0.106	− 0.340	− 0.587	− 0.264	− 0.57
SO_2_	0.078	0.160	− 0.306	− 0.053	− 0.454	− 0.420	− 0.239	− 0.368	− 0.032	0.080	− 0.074	− 0.27
O_3_	0.554	0.302	0.146	0.633	− 0.690	− 0.567	− 0.487	− 0.622	− 0.011	− 0.174	0.076	− 0.08

Temperature is positively correlated with O_3_ over all the cities with different values of coefficient. This correlation is relatively stronger in the spring and autumn seasons. Similar results were reported in previous studies [10, 56]. The rate of formation of O_3_ is higher when temperature increases, as photolytic activity is increased which helps in the formation of secondary pollutant O_3_ [11, 57]. The correlation among PM_2.5_ levels and temperature showed a weak negative trend during the autumn season over all the cities. However, the correlations are non-significant during other seasons. The PM_10_ levels showed a positive correlation with temperature during the spring and summer seasons, with the highest correlation (*R* = 0.43) found in Nanjing during the spring season. The mean NO_2_ levels showed weak negative correlations with temperature during the summer and autumn seasons over all the cities; whereas, the mean SO_2_ levels showed weak negative correlations with temperature during the winter and autumn season, and weak positive correlations during the spring and summer seasons. The relative humidity is negatively correlated with all the pollutants. The concentration of O_3_ showed a strong negative correlation with relative humidity. The NO_2_ and PM_2.5_ are weakly and negatively correlated with relative humidity. However, SO_2_ and PM_10_ are found to be moderate and negatively correlated. A similar trend is observed over all the cities.

Generally, the impact of the meteorological parameters on pollutant concentration is similar over all the cities of YRD. The increase in temperature and lower relative humidity favors the accumulation of O_3_, while low temperature, low wind speeds, and lower relative humidity favor the accumulation of primary pollutants.

### Monthly variations

**Fig. 7:**
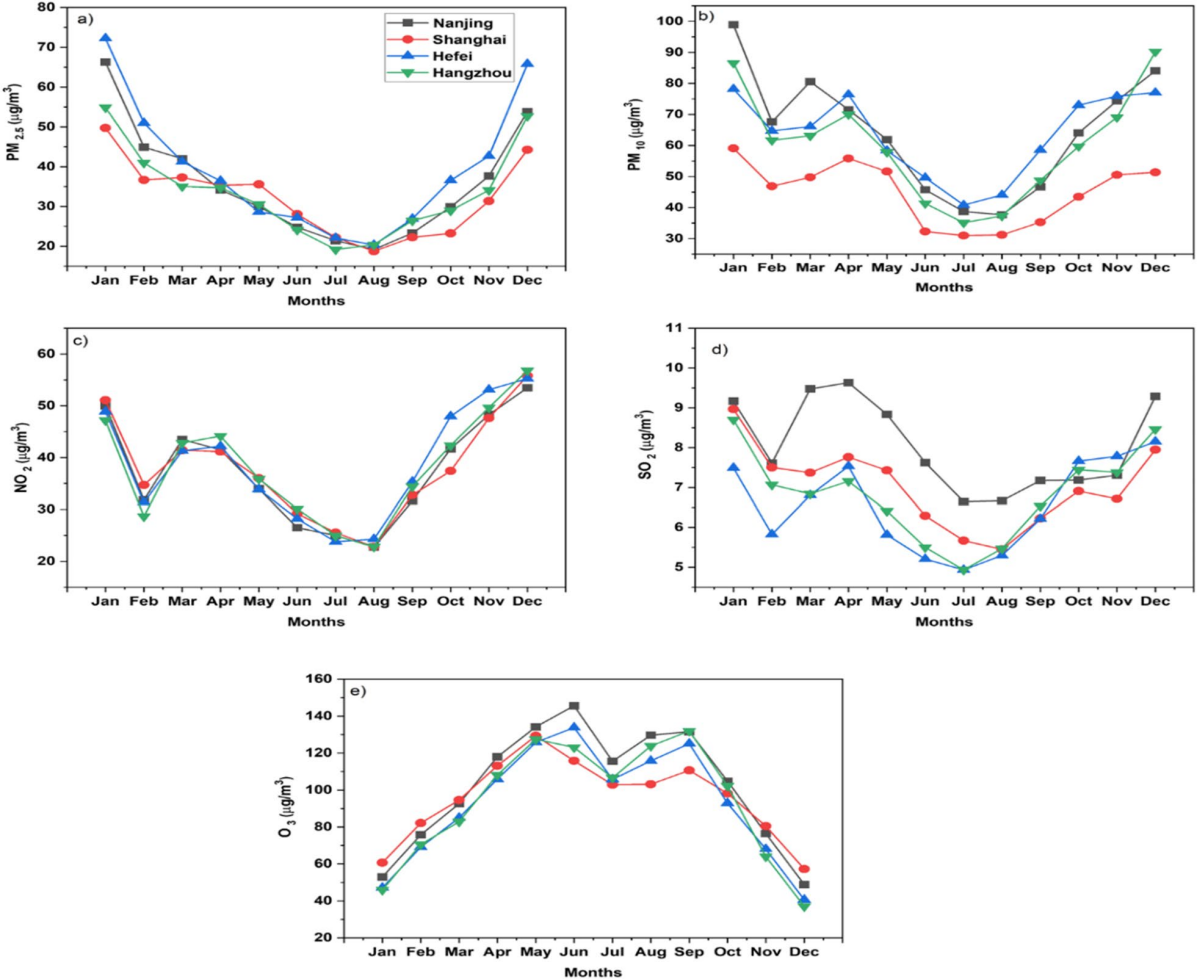
Multiyear (2018–2021) monthly average variations of **a** PM_2.5_, **b** PM_10_, **c** NO_2_, **d** SO_2_ and **e** O_3_ concentrations in the cities of the YRD region

The monthly variations in air pollutants over Shanghai, Nanjing, Hefei, and Hangzhou are investigated. Fig. 7 depicts multiyear (2018–2021) monthly variations in five criteria pollutants over the four cities. The results indicated that the pollutant concentrations are higher during the winter months for all the species (except O_3_). The concentration of pollutants began to rise in September and peaks were found in December–January. The peak values were observed during January for PM_2.5_ and PM_10_ except for Hangzhou where PM_10_ peaked during December. NO_2_ concentration peaked in December for all the cities while SO_2_ remained highest during January for Shanghai and Hangzhou, while for Nanjing and Hefei, it peaked during December. In contrast, O_3_ concentrations were observed to rise during the summer months with the highest value occurring in the May–June months.

The concentration of particulate matter peaked during the winter season due to different reasons. These reasons include stagnant meteorological conditions [lower temperature, lower wind speed] of locality [12], higher coal combustion for the heating purpose [58, 59], and formation of new particles and secondary organic–inorganic aerosols [12, 60]. Similarly, the concentration of SO_2_ and NO_2_ is also higher during winter months due to increased burning of biomass fuel coupled with reduced air temperature, wind speed, and solar radiations [36].

However, the concentration of O_3_ is higher in the summer months which can be attributed to the fact that photochemical reactions are its precursors. The monthly mean variation in the O_3_ levels shows an M-shaped curve, and the O_3_ peaks were observed in May and September for Shanghai and Hangzhou; whereas, in Hefei and Nanjing, the O_3_ peaks were observed in June and September. Similar monthly variations of O_3_ were reported in a study over south China [61]. The monthly variations of pollutants are consistent with findings of previous studies over different cities of China [12, 62].

There are a few anomalies in the results which are worth mentioning. The concentration of NO_2_, SO_2_, and PM_10_ showed a sudden decline in February. This can be linked to the fact that the Spring festival which is the most valuable vacation for Chinese people occurred in February during these years (Additional file 1: Table S8). The majority of migrant residents of megacities return to their hometowns. The closure of industries, offices, and educational institutes is observed over all the regions of China during the spring festival. Therefore, the reduction in anthropogenic activities results in a sudden dip in pollutants concentrations. Similar results were reported in previous studies explaining the impact of the spring festival on pollutants concentration [48, 53].

**Fig. 8:**
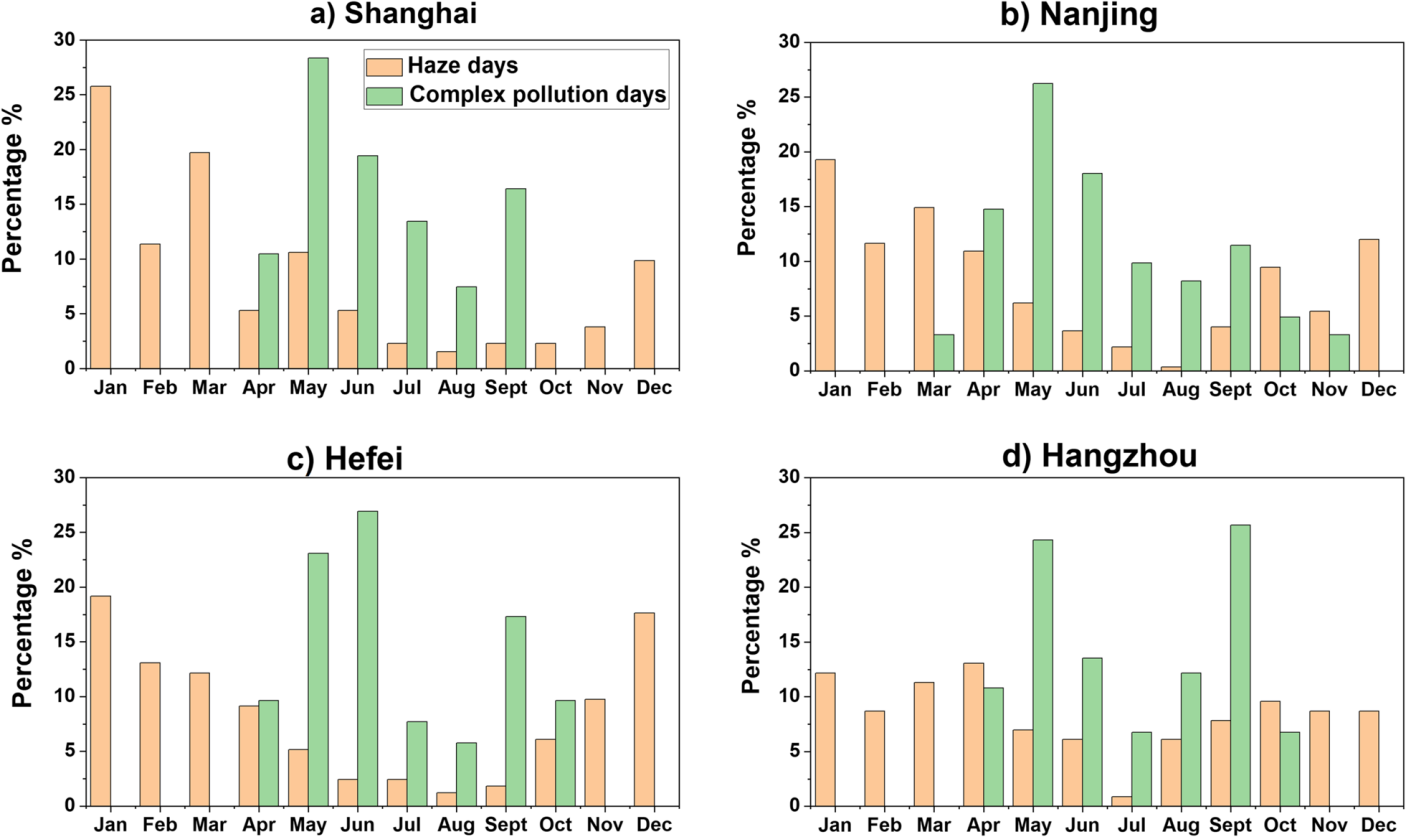
Multiyear (2018–2021) monthly variation in percentage (%) of haze and complex pollution days in the cities of the YRD region

Fig. 8 shows the percentage of haze and complex pollution days occurrence in different months from 2018 to 2021. It is observed that complex pollution days mainly occurred in summer, whereas the higher percentage of haze days occurred in winter. The maximum haze days occurred in January over all the cities. This occurrence of haze episodes in winter months can be attributed to the fact that fine particulates concentration is higher in winter which plays an important role in haze formation; whereas, the maximum complex pollution days occurred in May over Shanghai and Nanjing, in June over Hefei, and in September over Hangzhou. This distribution of complex days is mainly because O_3_ and PM_2.5_ are negatively correlated during winter and autumn. However, the correlation is moderate positive during late spring and summer [63, 64].

### TROPOMI validation

**Fig. 9:**
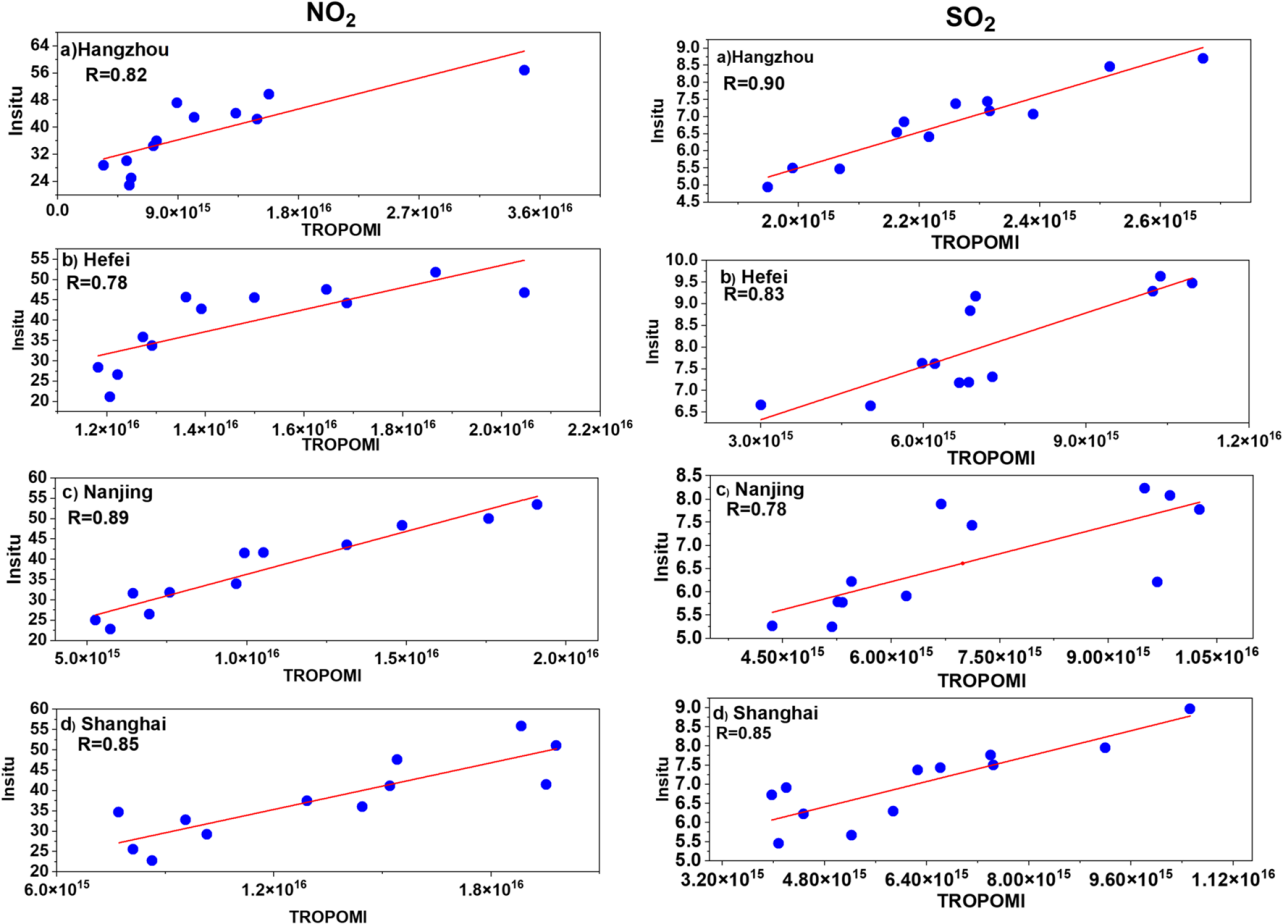
Scatter plot between TROPOMI and in situ measurement of multiyear (2018–2021) monthly mean data of SO_2_ and NO_2_ over **a** Hangzhou, **b** Hefei, **c** Nanjing and **d** Shanghai. The results show that there is strong correlation among both datasets over all the cities

The in situ measurements for trace gases including SO_2_ and NO_2_ are compared with spatially averaged TROPOMI satellite data of SO_2_ and NO_2_ over different cities of YRD region. The multiyear (2018–2021) monthly mean values of SO_2_ and NO_2_ are compared with each other. Fig. 9 shows correlation plot among in situ and TROPOMI satellite data.

### Principal component analysis and correlation analysis

PCA was used to reveal possible sources of pollutants emissions and calculate correlations between them. PCA is a frequently utilized multi-variate statistical analysis tool that is crucial to the study of interrelationships between diverse variables coming from a similar transference path or source [12, 33]. Additional file 1: Tables S9–S12 represents the total variance and rotated component matrix of PCA of atmospheric pollutants concentrations in our study area. Two factors were obtained which explain 83%, 81%, 80%, and 78% total variability over Hangzhou, Shanghai, Nanjing, and Hefei. Table 3 depicts the two factors obtained through PCA.

**Table 3:** Factor loadings after PCA analysis Varimax rotation

	Hefei			Nanjing			Shanghai			Hangzhou	
	Factor 1	Factor 2		Factor 1	Factor 2		Factor 1	Factor 2		Factor 1	Factor 2
PM_2.5_	0.744		PM_2.5_	0.867		PM_2.5_	0.886		PM_2.5_	0.926	
PM_10_	0.899		PM_10_	0.916		PM_10_	0.869		PM_10_	0.954	
SO_2_	0.819		SO_2_	0.756		SO_2_	0.854		SO_2_	0.814	
NO_2_	0.879		NO_2_	0.873		NO_2_	0.806		NO_2_	0.825	
O_3_		0.961	O_3_		0.953	O_3_		0.978	O_3_		0.990

**Fig. 10:**
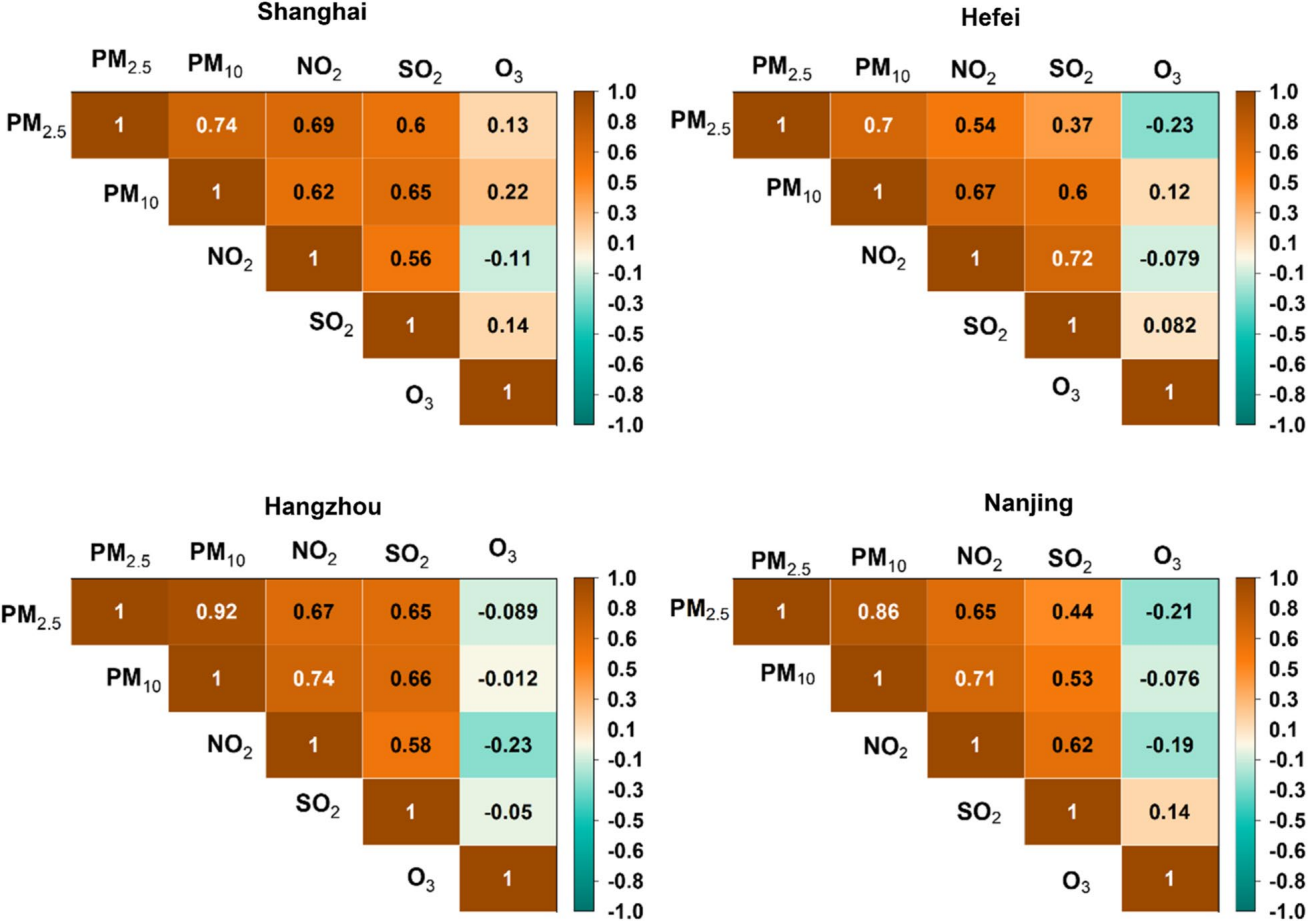
Correlation matrix of daily mean criteria pollutants from 2018 to 2021

Factor 1 has positive loading of SO_2_, PM_2.5_, PM_10_, and NO_2_. These all are indicative of primary anthropogenic sources (industrial, coal, biomass and fossil fuel). However, factor 2 has positive loading of O_3_ reflecting markedly unlike sources that are photochemical reactions. Similar results were observed over all the cities with different values. These results are consistent with findings over urban cities of Lanzhou, Urumqi, Jinan and Shanghai [12, 33, 65, 66]. It is worth mentioning that Lanzhou, Urumqi and Jinan are located at geographically different locations as compared to study area. Fig.10 depicts the correlation matrix of trace gases and particulate matter over cities of the YRD region.

The daily mean PM_2.5_ concentrations are strongly correlated with daily mean PM_10_ concentration in all the cities. The strongest correlation is observed over Hangzhou as compared to other cities. Particulate matter (PM_2.5_ and PM_10_) shows an almost similar correlation trend with NO_2_ and SO_2_ over all the cities of the study. The positive correlation between particulate matter (PM_2.5_ and PM_10_) with SO_2_ and NO_2_ is noted. This indicates that particulate matters (PM_2.5_ and PM_10_), and primary trace gases (SO_2_ and NO_2_) have similar sources of emissions like fossil fuel combustion and traffic. The recent studies also stated a strong positive correlation between particulate matters (PM_2.5_ and PM_10_), and primary trace gases in Beijing [67] and Delhi [68]. The correlation of O_3_ with other trace gases and particulate matter showed diverse trends over the different cities of this study. A weak positive correlation is observed between particulate matter (PM_2.5_ and PM_10_) and O_3_ over Shanghai; whereas, over Hangzhou and Nanjing this correlation among is particulate matters (PM_2.5_ and PM_10_) and O_3_ weak negative. PM_2.5_ is negatively correlated with O_3_ in Hefei, whereas PM_10_ showed a weak positive correlation. A weak negative correlation is observed among NO_2_ over all the cities. The correlation coefficient between SO_2_ and O_3_ is weak positive over Shanghai and Nanjing; whereas, the correlation coefficient between SO_2_ and O_3_ is weak negative over Hefei and Hangzhou.

The results depict that O_3_ had distinctly different sources as compared to primary trace gases and particulate matters. The negative correlation of O_3_ with NO_2_ can be explained by studying the tropospheric chemistry where the reaction of O_3_ with NO leads to the formation of NO_2_ thereby depleting O_3_ [69]. These results comply with previously reported findings [10, 55].

### Potential source contribution function

**Fig. 11:**
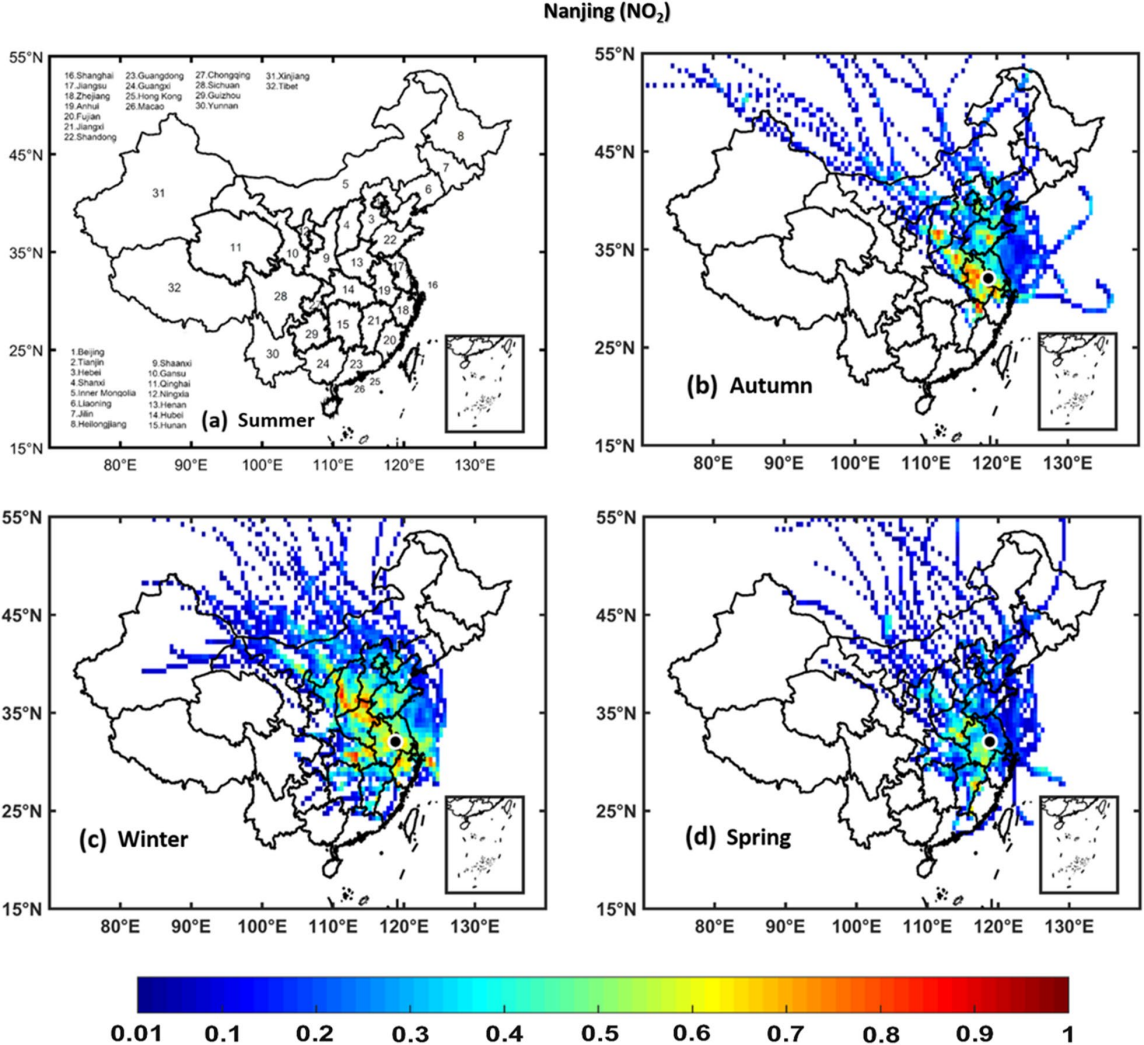
PSCF analysis based on NO_2_ grouped by season over Nanjing. The color bar indicates the weights of Pollution source regions

**Fig. 12:**
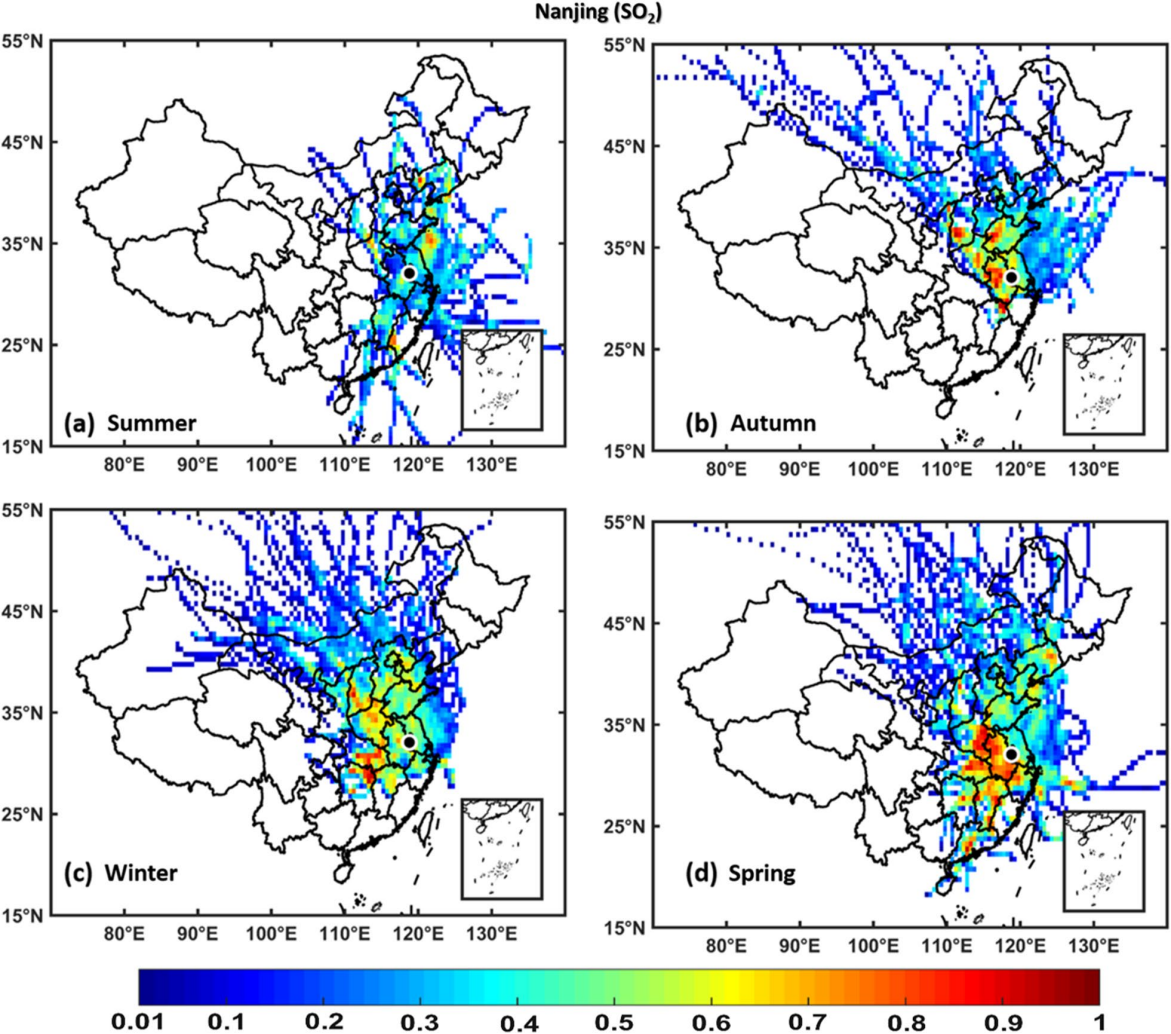
PSCF analysis based on SO_2_ grouped by season over Nanjing. The color bar indicates the weights of Pollution source regions

**Fig. 13:**
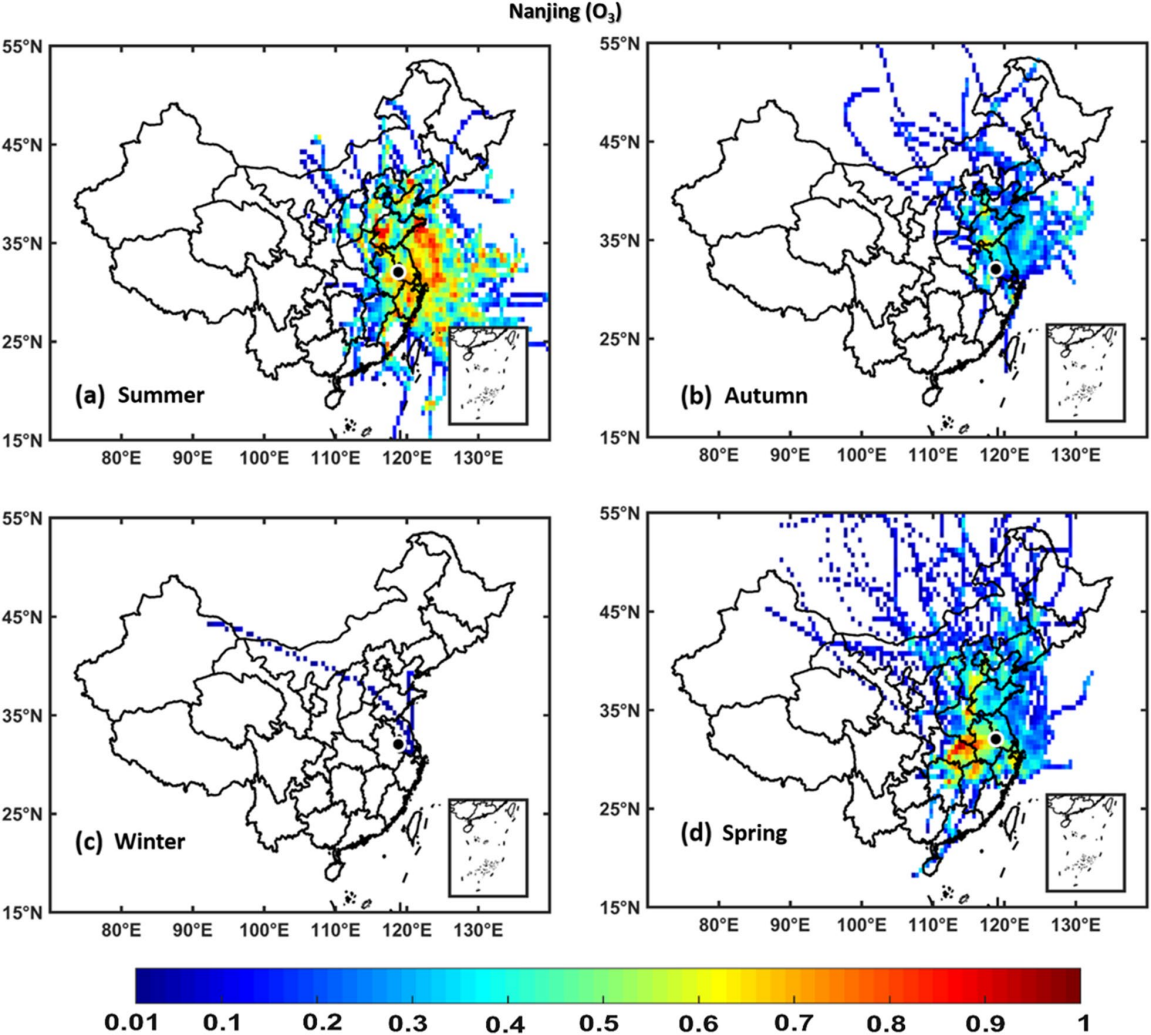
PSCF analysis based on O_3_ grouped by season over Nanjing. The color bar indicates the weights of pollution source regions

**Fig. 14:**
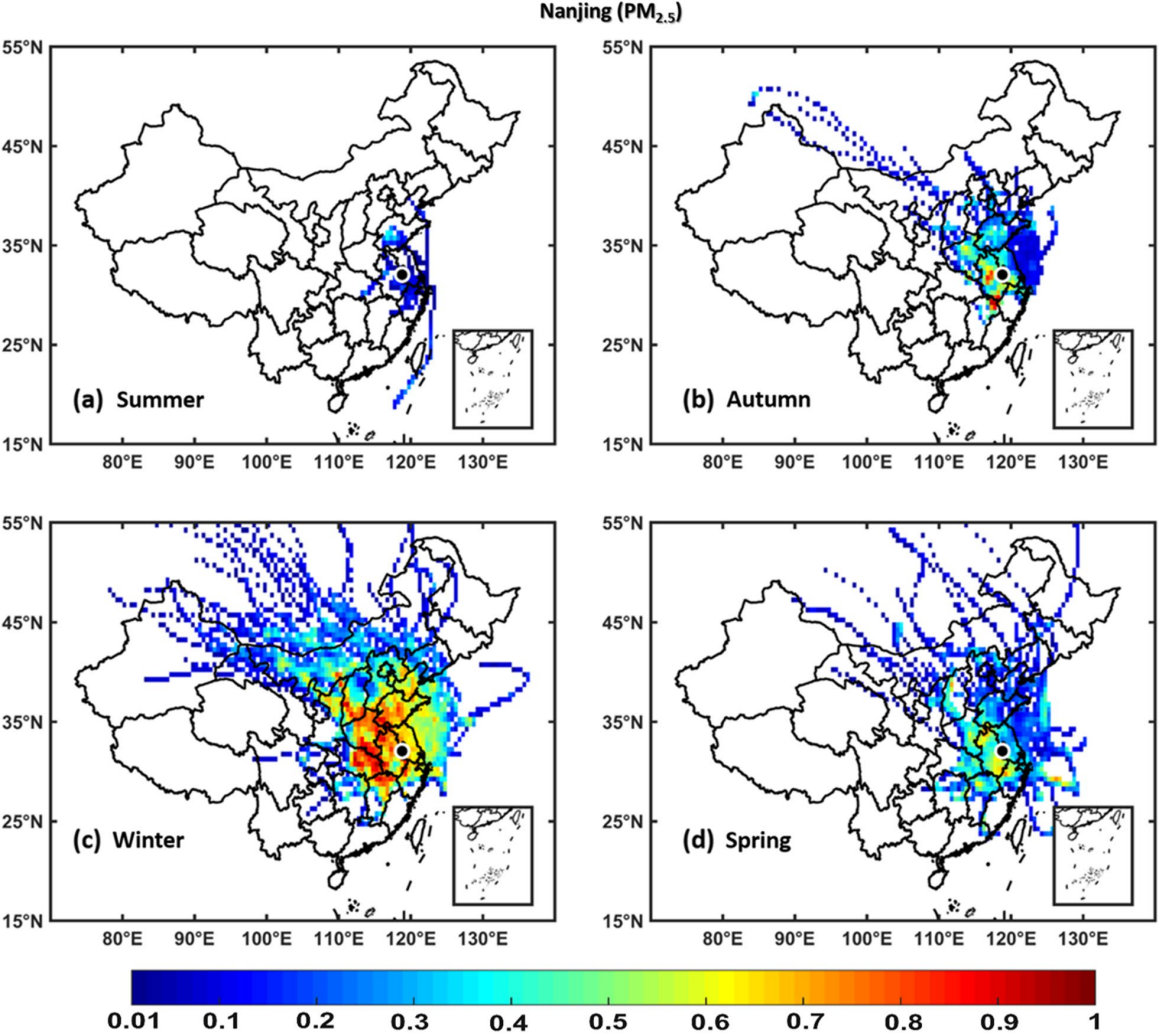
PSCF analysis based on PM_2.5_ grouped by season over Nanjing. The color bar indicates the weights of pollution source regions

**Fig. 15:**
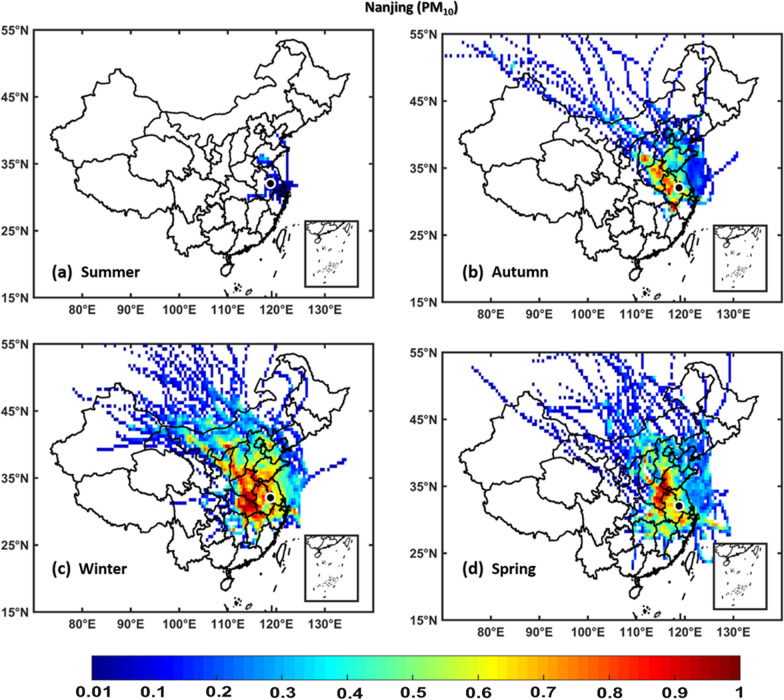
PSCF analysis based on PM_10_ grouped by season over Nanjing. The color bar indicates the weights of pollution source regions

PSCF analyses were performed to identify the potential source areas of NO_2_, SO_2_, O_3_, PM_2.5_, and PM_10_ over Hangzhou, Hefei, Nanjing, and Shanghai regions using seasonal in situ measurements of NO_2_, SO_2_, O_3_, PM_2.5_, and PM_10_ as input in the PSCF model and 72-h back trajectories obtained from the NOAA HYSPLIT model. The regions with WPSCF less than 0.4 are described as low pollution source, regions with WPSCF ranging from 0.4-0.5 are described as medium pollution source, whereas areas with WPSCF greater than 0.5 are termed as high pollution source regions. The results show that the air quality over Hangzhou, and Hefei, Nanjing, and Shanghai are significantly affected by local [neighboring provinces and cities within China] pollution sources of NO_2_, SO_2_, PM_2.5_, and PM_10_ pollutants, which are stronger in winter and weaker in summer, whereas an opposite scenario was observed for O_3_. Figs. 11, 12, 13, 14, 15 (Additional file 1: Figures S2–S16) show PSCF Analysis based on NO_2_, SO_2_, O_3_, PM_2.5_, and PM_10_, respectively grouped by season over Nanjing (Hefei, Hangzhou and Shanghai, respectively). Additional file 1: Figure S17 shows different regions in People’s republic of China Figs.12, 13, 14,15 .

Generally, the wintertime high values of PSCF (> 0.50) over Hangzhou, Hefei, Nanjing, and Shanghai regions indicate the stronger contributions from local source areas (Anhui, Beijing, Chongqing, Fujian, Hebei, Henan, Hubei, Hunan, Guangdong, Jiangsu, Jiangxi, Inner Mongolia, Shanxi, Shaanxi, Shandong, Sichuan, Tianjin, and Zhejiang). These local sources of PM_2.5_ concentrations more significantly affect the air quality of Hangzhou, Hefei, Nanjing, and Shanghai than regional sources. In spring, the most common local source areas (e.g., Anhui, Fujian, Hebei, Henan, Hubei, Hunan, Guangdong, Jiangsu, Jiangxi, Inner Mongolia, Shanxi, Shandong, and Zhejiang) of NO_2_, SO_2_, O_3_, PM_2.5_, and PM_10_ concentrations also substantially affect the air quality of Hangzhou, Hefei, Nanjing, and Shanghai.

The summertime air quality of these four study areas was significantly influenced by O_3_, whereas the lowest contributions were noted for NO_2_, SO_2_, PM_2.5_, and PM_10_ concentrations. This is showing the influence of local meteorology on aerosol pollutants and trace gases, i.e., more dispersion of aerosols and trace gases is occurred in summer than winter due to strong solar radiations, high temperatures leading to a high planetary boundary layer [36, 70]. However, the most common potential source areas of O_3_ concentrations are Anhui, Beijing, Fujian, Hebei, Henan, Hubei, Hunan, Guangdong, Jiangsu, Jiangxi, Shanxi, Shandong, Tianjin, and Zhejiang. In addition, the autumn air quality over Hangzhou, Hefei, Nanjing, and Shanghai are significantly impacted by local pollution sources. Overall, the results show that local sources from mainland China are the major contributors of air pollution over Hangzhou, Hefei, Nanjing, and Shanghai, with PM_2.5_ pollution levels being higher in winter than in other seasons, whereas O_3_ pollution is higher in summer.

## Conclusions

This study investigates the recent spatiotemporal variations (2018–2021) of trace gases and aerosols along with the impact of trans-boundary air contamination and the effect of meteorological parameters on air quality in the cities of YRD region (Shanghai, Hefei, Hangzhou and Nanjing). The annual mean concentration of NO_2_ and PM_2.5_ continuously declines from 2019 to 2021 over all the cities. The unprecedented decline in annual mean O_3_ concentration is observed in 2020 over the cities of YRD region. The concurrent reduction of both NO_x_ and VOCs during 2020 due to ongoing mitigation plan and new stringent control measures introduced by the Chinese Ministry of Environment and Ecology in 2020 may be helped in the reduction of O_3_ concentration. The results showed still particulate matters exceeded the NAAQS and WHO guidelines although strict measures have resulted in significant improvement of air quality. The Haze pollution events occurred more frequently in Hefei and Nanjing as compared to Shanghai and Hangzhou. This can be attributed to higher concentration of PM_2.5_ in Hefei and Nanjing. The complex pollution episodes, i.e., the concurrent occurrence of PM_2.5_ and O_3_ are higher in Shanghai and Hangzhou possibly due to higher atmospheric oxidation capacity which enhances the rate of secondary pollution formation. The primary pollutants concentrations were higher in the winter, whereas O_3_ levels were higher during the summer season. The higher concentration of trace gases and particulate matter were mainly due to trans-boundary transport from adjoining cities and provinces. The increase in temperature and lower relative humidity favors the accumulation of O_3_, while low temperature, low wind speeds, and lower relative humidity favor the accumulation of primary pollutants. Different cities have different pollution characteristics, requiring continuous monitoring to design city-specific policies and action plans. The results of this study can be used as a strong reference for the future research by scientific community, administration and policymakers, and other stakeholders who might be concerned about air pollution impacts and mitigation over this vast, populous and economically crucial region. Future emission control strategies should be designed considering the synchronized control of O_3_ and PM_2.5_, especially for the cities with higher atmospheric oxidation capacity.

## Supplementary Information

**Additional file 1:** **Table S1.** The spatial position of National monitoring stations over the studied cities of YRD region. **Table S2.** Criteria air pollutants in Hangzhou during the study period (2018-2021). **Table S3.** Criteria air pollutants in Nanjing during the study period (2018-2021). **Table S4.** Criteria air pollutants in Hefei during the study period (2018–2021). **Table S5.** Criteria air pollutants in Shanghai during the study period (2018–2021). **Table S6**. The standards for annual mean concentration (µg/m^3^) of different pollutants. **Table S7**. The relative (%) change in concentration of trace gases and particulate matter from 2019-2020. **Table S8.** Spring Festival during different Years in China. **Table S9.** Total variance and rotated component matrix of PCA of pollutants concentrations over Nanjing. **Table S10.** Total variance and rotated component matrix of PCA of pollutants concentrations over Hangzhou. **Table S11.** Total variance and rotated component matrix of PCA of pollutants concentrations over Shanghai. **Table S12.** Total variance and rotated component matrix of PCA of pollutants concentrations over Hefei. **Figure S1.** The inter- annual variation of NO_2_, O_3_, SO_2_, PM2.5 and PM10 in Hangzhou, Nanjing, Hefei and Shanghai during 2018-2021excluding COVID-19 lockdown days (24th of January–31st of March) from all years. **Figure S2.** PSCF Analysis based on NO_2_ grouped by season over Hefei. The color bar indicates the weights of Pollution source regions. **Figure S3.** PSCF Analysis based on SO_2_ grouped by season over Hefei. The color bar indicates the weights of Pollution source regions. **Figure S4. **PSCF Analysis based on O_3_ grouped by season over Hefei. The color bar indicates the weights of Pollution source regions. **Figure S5.** PSCF Analysis based on PM_2.5_ grouped by season over Hefei. The color bar indicates the weights of Pollution source regions. **Figure S6.** PSCF Analysis based on PM_10_ grouped by season over Hefei. The color bar indicates the weights of Pollution source regions. **Figure S7.** PSCF Analysis based on NO_2_ grouped by season over Hangzhou. The color bar indicates the weights of Pollution source regions. **Figure S8.** PSCF Analysis based on SO_2_ grouped by season over Hangzhou. The color bar indicates the weights of Pollution source regions. **Figure S9.** PSCF Analysis based on O_3_ grouped by season over Hangzhou. The color bar indicates the weights of Pollution source regions. **Figure S10.** PSCF Analysis based on PM_2.5_ grouped by season over Hangzhou. The color bar indicates the weights of Pollution source regions. **Figure S11.** PSCF Analysis based on PM_10_ grouped by season over Hangzhou. The color bar indicates the weights of Pollution source regions. **Figure S12.** PSCF Analysis based on NO_2_ grouped by season over Shanghai. The color bar indicates the weights of Pollution source regions. **Figure S13.** PSCF Analysis based on SO_2_ grouped by season over Shanghai. The color bar indicates the weights of Pollution source regions. **Figure S14.** PSCF Analysis based on O_3_ grouped by season over Shanghai. The color bar indicates the weights of Pollution source regions. **Figure S15.** PSCF Analysis based on PM_2.5_ grouped by season over Shanghai. The color bar indicates the weights of Pollution source regions. **Figure S16.** PSCF Analysis based on PM_10_ grouped by season over Shanghai. The color bar indicates the weights of Pollution source regions. **Figure S17.** Different regions of Peoples republic of China.

## Data Availability

The datasets used and/or analyzed during the current study are available from the corresponding author on reasonable request.

## Abbreviations

µg/m^3^: Microgram per cubic meter
AGL: Above ground level
AMF: Air mass factor
DOAS: Differential optical absorption spectroscopy
FGD: Fuel gas desulfurization
GDAS: Global data assimilation system
GDP: Gross domestic product
HYSPLIT: Hybrid single-particle Lagrangian integrated trajectory
MDA8 ozone: Maximum daily 8-hour average ozone
NAAQS: National Ambient Air Quality Standards
NCP: North China Plain
NO_2_: Nitrogen dioxide
NOAA: National Oceanic and Atmospheric Administration
O_3_: Ozone
PC: Principal component
PCA: Principal component analysis
PM: Particulate matter
PSCF: Potential source contribution function
SCD: Slant column density
SO_2_: Sulphur dioxide
TROPOMI: Tropospheric monitoring instrument
VCD: Vertical column density
VOCs: Volatile organic compounds
WHO: World Health Organization
WPSCF: Weighted PSCF
YRD: Yangtze River Delta
