# Supplementary material for: Spatiotemporal characterization of aerosols and trace gases over the Yangtze River Delta region, China: impact of trans-boundary pollution and meteorology
Source: Environ Sci Eur. 2022 Sep 8;34(1):86. doi: 10.1186/s12302-022-00668-2 (PMC9453706; doi:10.1186/s12302-022-00668-2)
Supplement: Supplementary file 1 — Additional file 1: Table S1. The spatial position of National monitoring stations over the studied cities of YRD region. Table S2. Criteria air pollutants in Hangzhou during the study period (2018-2021). Table S3. Criteria air pollutants in Nanjing during the study period (2018-2021). Table S4. Criteria air pollutants in Hefei during the study period (2018–2021). Table S5. Criteria air pollutants in Shanghai during the study period (2018–2021). Table S6. The standards for annual mean concentration (µg/m3) of different pollutants. Table S7. The relative (%) change in concentration of trace gases and particulate matter from 2019-2020. Table S8. Spring Festival during different Years in China. Table S9. Total variance and rotated component matrix of PCA of pollutants concentrations over Nanjing. Table S10. Total variance and rotated component matrix of PCA of pollutants concentrations over Hangzhou. Table S11. Total variance and rotated component matrix of PCA of pollutants concentrations over Shanghai. Table S12. Total variance and rotated component matrix of PCA of pollutants concentrations over Hefei. Figure S1. The inter- annual variation of NO2, O3, SO2, PM2.5 and PM10 in Hangzhou, Nanjing, Hefei and Shanghai during 2018-2021excluding COVID-19 lockdown days (24th of January–31st of March) from all years. Figure S2. PSCF Analysis based on NO2 grouped by season over Hefei. The color bar indicates the weights of Pollution source regions. Figure S3. PSCF Analysis based on SO2 grouped by season over Hefei. The color bar indicates the weights of Pollution source regions. Figure S4. PSCF Analysis based on O3 grouped by season over Hefei. The color bar indicates the weights of Pollution source regions. Figure S5. PSCF Analysis based on PM2.5 grouped by season over Hefei. The color bar indicates the weights of Pollution source regions. Figure S6. PSCF Analysis based on PM10 grouped by season over Hefei. The color bar indicates the weights of Pollution source regions. Figur [file 12302_2022_668_MOESM1_ESM.docx]

**Additional file 1**

**Spatiotemporal Characterization of Aerosols and Trace Gases over the Yangtze River Delta Region, China: Impact of transboundary pollution and meteorology**

Zeeshan Javed ^1, †^, Muhammad Bilal ^2, †^, Zhongfeng Qiu ^2^, Guanlin Li ^1^, Osama Sandhu ^3^, Khalid Mehmood ^4^, Yu Wang ^2^, Md. Arfan Ali ^2^, Cheng Liu ^5,6,7,8^, Yuhang Wang ^9^, Ruibin Xue ^10^, Daolin Du ^1^, * and Xiaojun Zheng ^1,^ *

**Table S1**

The spatial position of National monitoring stations over the studied cities of YRD region.

| City | Observation site | Longitude | Latitude |
| --- | --- | --- | --- |
| Shanghai | Putuo | 121.4 | 31.238 |
|  | Fifteenth Factory | 121.478 | 31.2036 |
|  | Hongkou | 121.467 | 31.3008 |
|  | Xuhui upper normal university | 121.412 | 31.1654 |
|  | Yangpu four drifts | 121.536 | 31.2659 |
|  | Qingpu dianshan lake | 120.978 | 31.0935 |
|  | Jing'an monitoring station | 121.425 | 31.2261 |
|  | Pudong chuansha | 121.703 | 31.1907 |
|  | Pudong new area | 121.533 | 31.2284 |
|  | Pudong zhangjiang | 121.577 | 31.2071 |
| Nanjing | Maigao bridge | 118.803 | 32.1083 |
|  | Pasture gate | 118.749 | 32.0572 |
|  | Shanxi road | 118.778 | 32.0723 |
|  | China gate | 118.777 | 32.0144 |
|  | Ruijin road | 118.803 | 32.0314 |
|  | Xuanwu lake | 118.795 | 32.0775 |
|  | Pukou | 118.626 | 32.0878 |
|  | Olympic sports center | 118.737 | 32.0092 |
|  | Xianlin university town | 118.907 | 32.105 |
| Hangzhou | Riverside | 120.211 | 30.21 |
|  | Xixi | 120.063 | 30.2747 |
|  | Xiasha | 120.348 | 30.3058 |
|  | Wolong bridge | 120.127 | 30.2456 |
|  | Zhejiang agricultural university | 120.19 | 30.2692 |
|  | Zhaohui district five | 120.157 | 30.2897 |
|  | Harmony primary school | 120.12 | 30.3119 |
|  | Linping town | 120.301 | 30.4183 |
|  | Chengxiang town | 120.27 | 30.1819 |
|  | Yunqi | 120.088 | 30.1808 |
| Hefei | Pearl plaza | 117.196 | 31.7848 |
|  | Sanli street | 117.307 | 31.8766 |
|  | Amber hill | 117.259 | 31.8706 |
|  | Dongpu reservoir | 117.16 | 31.9051 |
|  | Changjiang middle road | 117.25 | 31.8572 |
|  | Luyang district | 117.266 | 31.9438 |
|  | Yaohai district | 117.336 | 31.8585 |
|  | Baohe district | 117.302 | 31.7956 |
|  | Binhu new district | 117.278 | 31.7386 |
|  | High-tech Zone | 117.124 | 31.8516 |

**Table S2**

Criteria air pollutants in Hangzhou during the study period (2018-2021).

| Pollutant | Unit | Mean | SD | Min | Max | Median |
| --- | --- | --- | --- | --- | --- | --- |
| **2018** |  |  |  |  |  |  |
| PM_2.5_ | µg/m^3^ | 37.8 | 23 | 6 | 139 | 31 |
| PM_10_ | µg/m^3^ | 64.4 | 35 | 13.8 | 193 | 54 |
| SO_2_ | µg/m^3^ | 8.41 | 2 | 5 | 22 | 8 |
| NO_2_ | µg/m^3^ | 39.66 | 15 | 11.1 | 100 | 38 |
| O_3_ | µg/m^3^ | 91.47 | 51 | 5 | 243 | 83 |
| **2019** |  |  |  |  |  |  |
| PM_2.5_ | µg/m^3^ | 38 | 21 | 6 | 135 | 33 |
| PM_10_ | µg/m^3^ | 66 | 34 | 8 | 209 | 61 |
| SO_2_ | µg/m^3^ | 06 | 02 | 3 | 17 | 07 |
| NO_2_ | µg/m^3^ | 41 | 15 | 7 | 89 | 41 |
| O_3_ | µg/m^3^ | 99 | 55 | 4 | 236 | 90 |
| **2020** |  |  |  |  |  |  |
| PM_2.5_ | µg/m^3^ | 29 | 17 | 6 | 115 | 26 |
| PM_10_ | µg/m^3^ | 55 | 29 | 8 | 187 | 49 |
| SO_2_ | µg/m^3^ | 06 | 1.48 | 4 | 12 | 6 |
| NO_2_ | µg/m^3^ | 38 | 15 | 7 | 88 | 36 |
| O_3_ | µg/m^3^ | 90 | 45 | 4 | 231 | 88 |
| **2021** |  |  |  |  |  |  |
| PM_2.5_ | µg/m^3^ | 28 | 14 | 5 | 100 | 25 |
| PM_10_ | µg/m^3^ | 54 | 26 | 9 | 169 | 49 |
| SO_2_ | µg/m^3^ | 06 | 1.8 | 04 | 13 | 06 |
| NO_2_ | µg/m^3^ | 34 | 14 | 06 | 75 | 31 |
| O_3_ | µg/m^3^ | 95 | 46 | 10 | 258 | 85 |

CI: confidence interval (at 95% confidence level); SD: standard deviation.

**Table S3**

Criteria air pollutants in Nanjing during the study period (2018-2021).

| Pollutant | Unit | Mean | SD | Min | Max | Median |
| --- | --- | --- | --- | --- | --- | --- |
| **2018** |  |  |  |  |  |  |
| PM_2.5_ | µg/m^3^ | 35 | 24 | 6 | 217 | 29 |
| PM_10_ | µg/m^3^ | 63 | 37 | 10 | 365 | 55 |
| SO_2_ | µg/m^3^ | 08 | 4 | 1 | 26 | 7 |
| NO_2_ | µg/m^3^ | 37 | 16 | 06 | 103 | 33 |
| O_3_ | µg/m^3^ | 103 | 47 | 08 | 244 | 99 |
| **2019** |  |  |  |  |  |  |
| PM_2.5_ | µg/m^3^ | 40 | 24 | 5 | 145 | 34 |
| PM_10_ | µg/m^3^ | 71 | 39 | 12 | 365 | 62 |
| SO_2_ | µg/m^3^ | 10 | 3.5 | 4 | 24 | 09 |
| NO_2_ | µg/m^3^ | 41 | 16 | 10 | 96 | 39 |
| O_3_ | µg/m^3^ | 106 | 51 | 8 | 244 | 101 |
| **2020** |  |  |  |  |  |  |
| PM_2.5_ | µg/m^3^ | 31 | 20 | 6 | 133 | 27 |
| PM_10_ | µg/m^3^ | 56 | 29 | 11 | 168 | 50 |
| SO_2_ | µg/m^3^ | 07 | 2 | 2 | 17 | 6 |
| NO_2_ | µg/m^3^ | 35 | 15 | 13 | 103 | 33 |
| O_3_ | µg/m^3^ | 102 | 45 | 9 | 222 | 99 |
| **2021** |  |  |  |  |  |  |
| PM_2.5_ | µg/m^3^ | 29 | 17 | 3 | 91 | 25 |
| PM_10_ | µg/m^3^ | 59 | 34 | 6 | 314 | 54 |
| SO_2_ | µg/m^3^ | 5.5 | 1.8 | 3 | 14 | 05 |
| NO_2_ | µg/m^3^ | 32 | 16 | 6 | 87 | 29 |
| O_3_ | µg/m^3^ | 103 | 45 | 11 | 238 | 96 |

CI: confidence interval (at 95% confidence level); SD: standard deviation

**Table S4**

Criteria air pollutants in Hefei during the study period (2018-2021).

| Pollutant | Unit | Mean | SD | Min | Max | Median |
| --- | --- | --- | --- | --- | --- | --- |
| **2018** |  |  |  |  |  |  |
| PM_2.5_ | µg/m^3^ | 43 | 31 | 7 | 181 | 36 |
| PM_10_ | µg/m^3^ | 66 | 35 | 11 | 236 | 60 |
| SO_2_ | µg/m^3^ | 6.5 | 3 | 2 | 25 | 06 |
| NO_2_ | µg/m^3^ | 39 | 17 | 09 | 95 | 35 |
| O_3_ | µg/m^3^ | 94 | 43 | 10 | 216 | 90 |
| **2019** |  |  |  |  |  |  |
| PM_2.5_ | µg/m^3^ | 44 | 27 | 5 | 166 | 37 |
| PM_10_ | µg/m^3^ | 72 | 35 | 14 | 308 | 67 |
| SO_2_ | µg/m^3^ | 7 | 3.1 | 2 | 16 | 5.5 |
| NO_2_ | µg/m^3^ | 41 | 17 | 11 | 93 | 38 |
| O_3_ | µg/m^3^ | 103 | 49 | 4 | 220 | 98 |
| **2020** |  |  |  |  |  |  |
| PM_2.5_ | µg/m^3^ | 36 | 23 | 7 | 138 | 31 |
| PM_10_ | µg/m^3^ | 58 | 28 | 12 | 200 | 54 |
| SO_2_ | µg/m^3^ | 06 | 2 | 3 | 17 | 6 |
| NO_2_ | µg/m^3^ | 38 | 17 | 10 | 94 | 34 |
| O_3_ | µg/m^3^ | 89 | 40 | 6 | 229 | 88 |
| **2021** |  |  |  |  |  |  |
| PM_2.5_ | µg/m^3^ | 31 | 21 | 6 | 134 | 26 |
| PM_10_ | µg/m^3^ | 60 | 34 | 8 | 178 | 56 |
| SO_2_ | µg/m^3^ | 07 | 02 | 04 | 15 | 06 |
| NO_2_ | µg/m^3^ | 35 | 17 | 09 | 93 | 31 |
| O_3_ | µg/m^3^ | 90 | 40 | 07 | 218 | 84 |

CI: confidence interval (at 95% confidence level); SD: standard deviation

**Table S5**

Criteria air pollutants in Shanghai during the study period (2018-2021).

| Pollutant | Unit | Mean | SD | Min | Max | Median |
| --- | --- | --- | --- | --- | --- | --- |
| **2018** |  |  |  |  |  |  |
| PM_2.5_ | µg/m^3^ | 34 | 25 | 8 | 191 | 27 |
| PM_10_ | µg/m^3^ | 47 | 27 | 10 | 185 | 40 |
| SO_2_ | µg/m^3^ | 09 | 03 | 4 | 28 | 08 |
| NO_2_ | µg/m^3^ | 38 | 18 | 09 | 108 | 35 |
| O_3_ | µg/m^3^ | 96 | 40 | 19 | 249 | 88 |
| **2019** |  |  |  |  |  |  |
| PM_2.5_ | µg/m^3^ | 35 | 21 | 6 | 122 | 30 |
| PM_10_ | µg/m^3^ | 49 | 28 | 10 | 212 | 40 |
| SO_2_ | µg/m^3^ | 7 | 2 | 4 | 16 | 6 |
| NO_2_ | µg/m^3^ | 42 | 18 | 6 | 115 | 39 |
| O_3_ | µg/m^3^ | 98 | 41 | 16 | 274 | 91 |
| **2020** |  |  |  |  |  |  |
| PM_2.5_ | µg/m^3^ | 31 | 21 | 8 | 156 | 25 |
| PM_10_ | µg/m^3^ | 41 | 19 | 7 | 124 | 36 |
| SO_2_ | µg/m^3^ | 06 | 2 | 4 | 13 | 6 |
| NO_2_ | µg/m^3^ | 37 | 14 | 13 | 97 | 34 |
| O_3_ | µg/m^3^ | 97 | 38 | 14 | 244 | 92 |
| **2021** |  |  |  |  |  |  |
| PM_2.5_ | µg/m^3^ | 26 | 15 | 7 | 101 | 23 |
| PM_10_ | µg/m^3^ | 42 | 23 | 8 | 195 | 37 |
| SO_2_ | µg/m^3^ | 5.5 | 1.5 | 04 | 14 | 05 |
| NO_2_ | µg/m^3^ | 34 | 16.5 | 04 | 113 | 31 |
| O_3_ | µg/m^3^ | 92 | 53 | 16 | 207 | 207 |

CI: confidence interval (at 95% confidence level); SD: standard deviation

**Table S6**

The standards for annual mean concentration (µg/m^3^) of different pollutants.

| Pollutant | China | | WHO |
| --- | --- | --- | --- |
|  | **Grade-1** | **Grade-2** |  |
| NO_2_ | 40 | 40 | 40 |
| SO_2_ | 20 | 60 |  |
| PM_2.5_ | 15 | 35 | 10 |
| PM_10_ | 40 | 70 | 20 |

**Table S7**

The relative (%) change in concentration of trace gases and particulate matter from 2019-2020.

| Specie |  | | Hangzhou | Hefei | Nanjing | Shanghai |
| --- | --- | --- | --- | --- | --- | --- |
| NO_2_ | **Change in %** | **Total** | **₋7.32** | **₋7.3** | **₋14.6** | **₋11.9** |
|  |  | **Excluding lockdown Period** | **₋2.4** | **₋1.1** | **₋9.5** | **₋5.4** |
| PM_2.5_ | **Change in %** | **Total** | **₋23.7** | **₋18.1** | **₋22.5** | **₋11.4** |
|  |  | **Excluding lockdown Period** | **₋16.6** | **₋10.1** | **₋15.3** | **₋6.96** |
| PM_10_ | **Change in %** | **Total** | **₋13.63** | **₋19.71** | **₋20.8** | **₋16.3** |
|  |  | **Excluding lockdown Period** | **₋12.5** | **₋14.4** | **₋14.9** | **₋8.7** |
| O_3_ | **Change in %** | **Total** | **₋9** | **­₋13.5** | **₋3.77** | **₋1** |
|  |  | **Excluding lockdown Period** | **₋10.57** | **₋14.1** | **₋6.5** | **₋2** |
| SO_2_ | **Change in %** | **Total** | **0** | **₋14.2** | **₋30** | **₋14.3** |
|  |  | **Excluding lockdown Period** | **₋7.7** | **₋10** | **₋28** | **₋6.2** |

**Table S8**

Spring Festival during different Years in China.

| Year | Spring Festival |
| --- | --- |
| 2018 | 13 February-20 February |
| 2019 | 4 February–10 February |
| 2020 | 25 January– 31 January |
| 2021 | 11 February–17 February |

**Table S9**

Total variance and rotated component matrix of PCA of pollutants concentrations over Nanjing.

| **Component** | **Initial Eigenvalues** | | | **Extraction Sums of Squared Loadings** | | | **Rotation Sums of Squared Loadings** | | |
| --- | --- | --- | --- | --- | --- | --- | --- | --- | --- |
|  | **Total** | **% of Variance** | **Cumulative %** | **Total** | **% Of Variance** | **Cumulative %** | **Total** | **% Of Variance** | **Cumulative %** |
| 1 | 2.945 | 58.908 | 58.908 | 2.945 | 58.908 | 58.908 | 2.933 | 58.661 | 58.661 |
| 2 | 1.104 | 22.074 | 80.982 | 1.104 | 22.074 | 80.982 | 1.116 | 22.321 | 80.982 |
| 3 | .561 | 11.220 | 92.202 |  |  |  |  |  |  |
| 4 | .273 | 5.465 | 97.667 |  |  |  |  |  |  |
| 5 | .117 | 2.333 | 100.000 |  |  |  |  |  |  |
| Extraction Method: Principal Component Analysis. | | | |  |  |  |  |  |  |

**Table S10**

Total variance and rotated component matrix of PCA of pollutants concentrations over Hangzhou.

| **Component** | **Initial Eigenvalues** | | | **Extraction Sums of Squared Loadings** | | | **Rotation Sums of Squared Loadings** | | |
| --- | --- | --- | --- | --- | --- | --- | --- | --- | --- |
|  | **Total** | **% of Variance** | **Cumulative %** | **Total** | **% Of Variance** | **Cumulative %** | **Total** | **% Of Variance** | **Cumulative %** |
| 1 | 3.141 | 62.827 | 62.827 | 3.141 | 62.827 | 62.827 | 3.113 | 62.253 | 62.253 |
| 2 | 1.027 | 20.533 | 83.360 | 1.027 | 20.533 | 83.360 | 1.055 | 21.107 | 83.360 |
| 3 | .432 | 8.648 | 92.008 |  |  |  |  |  |  |
| 4 | .335 | 6.703 | 98.712 |  |  |  |  |  |  |
| 5 | .064 | 1.288 | 100.000 |  |  |  |  |  |  |
| Extraction Method: Principal Component Analysis. | | | |  |  |  |  |  |  |

**Table S11**

Total variance and rotated component matrix of PCA of pollutants concentrations over Shanghai.

| **Component** | **Initial Eigenvalues** | | | **Extraction Sums of Squared Loadings** | | | | **Rotation Sums of Squared Loadings** | | |
| --- | --- | --- | --- | --- | --- | --- | --- | --- | --- | --- |
|  | **Total** | **% of Variance** | **Cumulative %** | **Total** | | **% Of Variance** | **Cumulative %** | **Total** | **% Of Variance** | **Cumulative %** |
| 1 | 2.948 | 58.956 | 58.956 | 2.948 | 58.956 | | 58.956 | 2.924 | 58.482 | 58.482 |
| 2 | 1.068 | 21.361 | 80.317 | 1.068 | 21.361 | | 80.317 | 1.092 | 21.836 | 80.317 |
| 3 | .439 | 8.782 | 89.099 |  |  | |  |  |  |  |
| 4 | .305 | 6.096 | 95.195 |  |  | |  |  |  |  |
| 5 | .240 | 4.805 | 100.000 |  |  | |  |  |  |  |
| Extraction Method: Principal Component Analysis. | | | |  |  | |  |  |  |  |

**Table S12**

Total variance and rotated component matrix of PCA of pollutants concentrations over Hefei.

| **Component** | **Initial Eigenvalues** | | | **Extraction Sums of Squared Loadings** | | | **Rotation Sums of Squared Loadings** | | |
| --- | --- | --- | --- | --- | --- | --- | --- | --- | --- |
|  | **Total** | **% of Variance** | **Cumulative %** | **Total** | **% Of Variance** | **Cumulative %** | **Total** | **% Of Variance** | **Cumulative %** |
| 1 | 2.817 | 56.332 | 56.332 | 2.817 | 56.332 | 56.332 | 2.804 | 56.087 | 56.087 |
| 2 | 1.121 | 22.419 | 78.751 | 1.121 | 22.419 | 78.751 | 1.133 | 22.664 | 78.751 |
| 3 | .636 | 12.717 | 91.468 |  |  |  |  |  |  |
| 4 | .243 | 4.862 | 96.330 |  |  |  |  |  |  |
| 5 | .183 | 3.670 | 100.000 |  |  |  |  |  |  |
| Extraction Method: Principal Component Analysis. | | | |  |  |  |  |  |  |

**Figure Captions**

**Fig. S1.** The inter- annual variation of NO_2_, O_3_, SO_2_, PM2.5 and PM10 in Hangzhou, Nanjing, Hefei and Shanghai during 2018-2021excluding COVID-19 lockdown days (24^th^ of January-31^st^ of March) from all years.

**Fig. S2.** PSCF Analysis based on NO_2_ grouped by season over Hefei. The color bar indicates the weights of Pollution source regions.

**Fig. S3.** PSCF Analysis based on SO_2_ grouped by season over Hefei. The color bar indicates the weights of Pollution source regions.

**Fig. S4.** PSCF Analysis based on O_3_ grouped by season over Hefei. The color bar indicates the weights of Pollution source regions.

**Fig. S5.** PSCF Analysis based on PM_2.5_ grouped by season over Hefei. The color bar indicates the weights of Pollution source regions.

**Fig. S6.** PSCF Analysis based on PM_10_ grouped by season over Hefei. The color bar indicates the weights of Pollution source regions.

**Fig. S7.** PSCF Analysis based on NO_2_ grouped by season over Hangzhou. The color bar indicates the weights of Pollution source regions.

**Fig. S8.** PSCF Analysis based on SO_2_ grouped by season over Hangzhou. The color bar indicates the weights of Pollution source regions.

**Fig. S9.** PSCF Analysis based on O_3_ grouped by season over Hangzhou. The color bar indicates the weights of Pollution source regions.

**Fig. S10.** PSCF Analysis based on PM_2.5_ grouped by season over Hangzhou. The color bar indicates the weights of Pollution source regions.

**Fig. S11.** PSCF Analysis based on PM_10_ grouped by season over Hangzhou. The color bar indicates the weights of Pollution source regions.

**Fig. S12.** PSCF Analysis based on NO_2_ grouped by season over Shanghai. The color bar indicates the weights of Pollution source regions.

**Fig. S13.** PSCF Analysis based on SO_2_ grouped by season over Shanghai. The color bar indicates the weights of Pollution source regions.

**Fig. S14.** PSCF Analysis based on O_3_ grouped by season over Shanghai. The color bar indicates the weights of Pollution source regions.

**Fig. S15.** PSCF Analysis based on PM_2.5_ grouped by season over Shanghai. The color bar indicates the weights of Pollution source regions.

**Fig. S16.** PSCF Analysis based on PM_10_ grouped by season over Shanghai. The color bar indicates the weights of Pollution source regions.

**Fig. S17.** Different regions of Peoples republic of China.


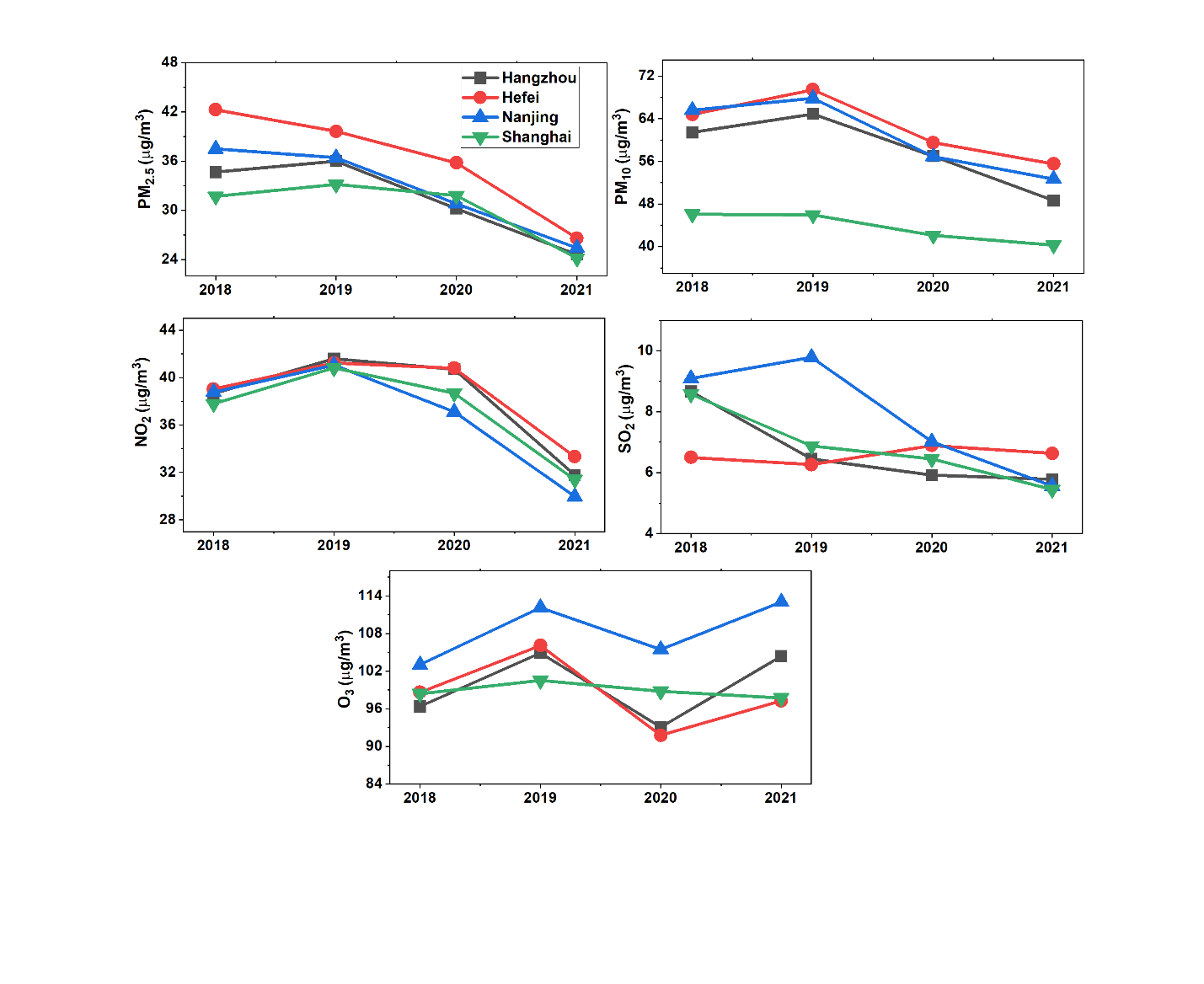


**Fig. S1.** The inter- annual variation of NO_2_, O_3_, SO_2_, PM2.5 and PM10 in Hangzhou, Nanjing, Hefei and Shanghai during 2018-2021excluding COVID-19 lockdown days (24^th^ of January-31^st^ of March) from all years.


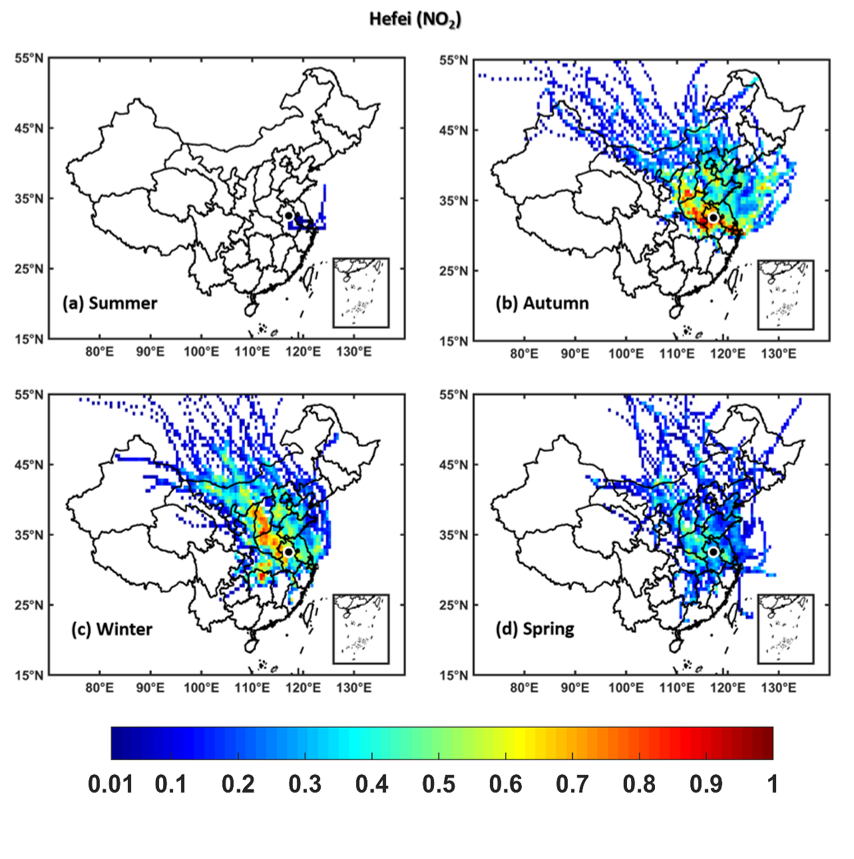


**Fig. S2.** PSCF Analysis based on NO_2_ grouped by season over Hefei. The color bar indicates the weights of Pollution source regions.


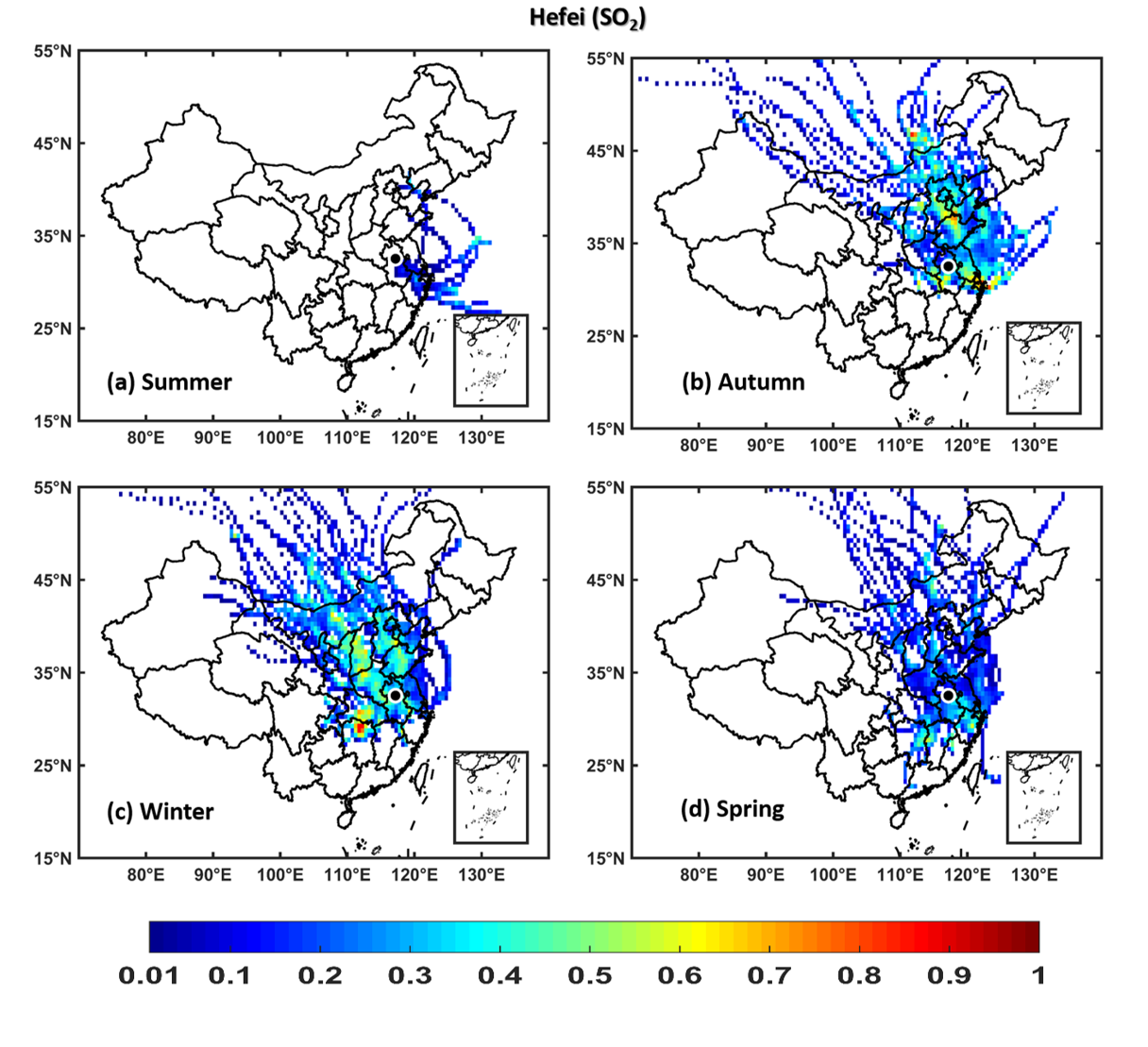


**Fig. S3.** PSCF Analysis based on SO_2_ grouped by season over Hefei. The color bar indicates the weights of Pollution source regions.


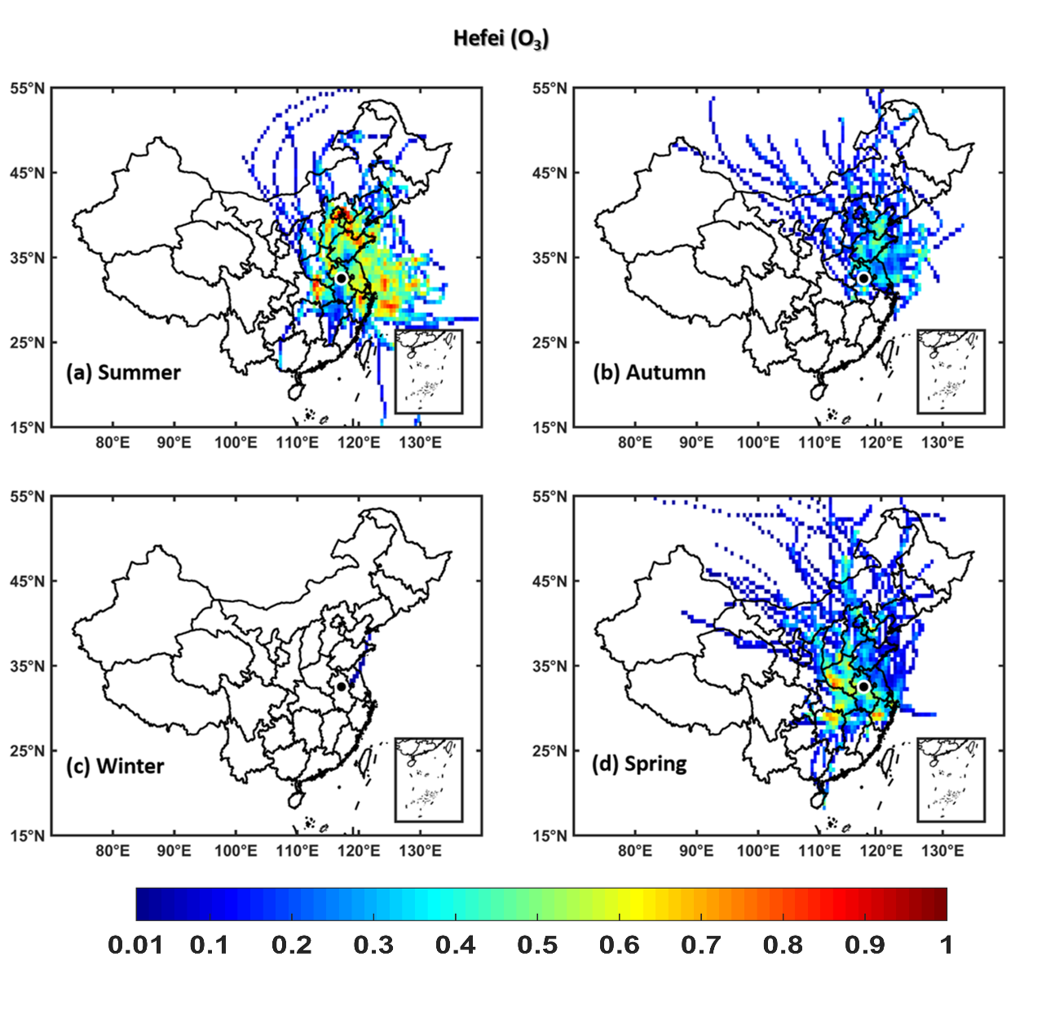


**Fig. S4.** PSCF Analysis based on O_3_ grouped by season over Hefei. The color bar indicates the weights of Pollution source regions.


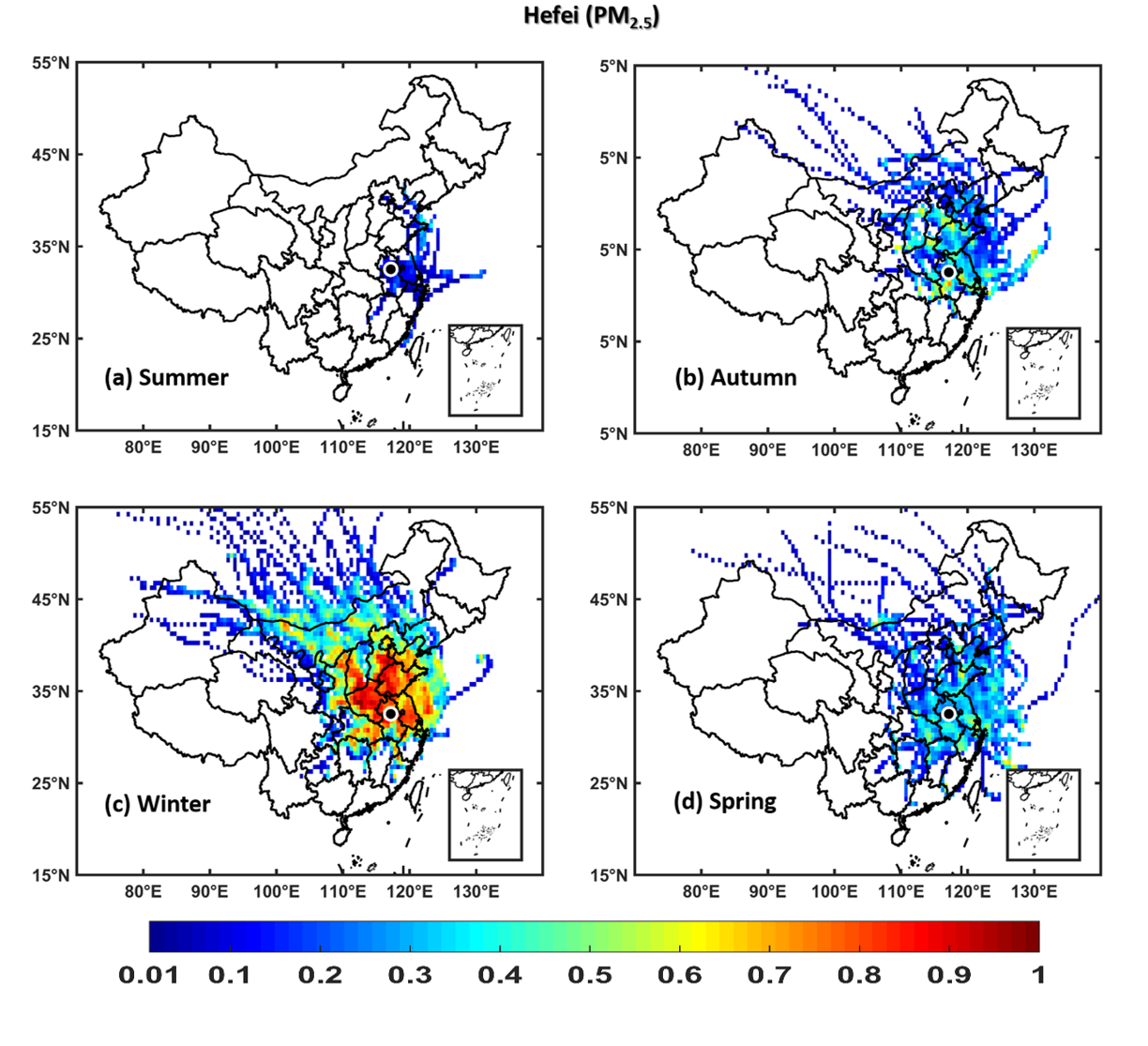


**Fig. S5.** PSCF Analysis based on PM_2.5_ grouped by season over Hefei. The color bar indicates the weights of Pollution source regions.


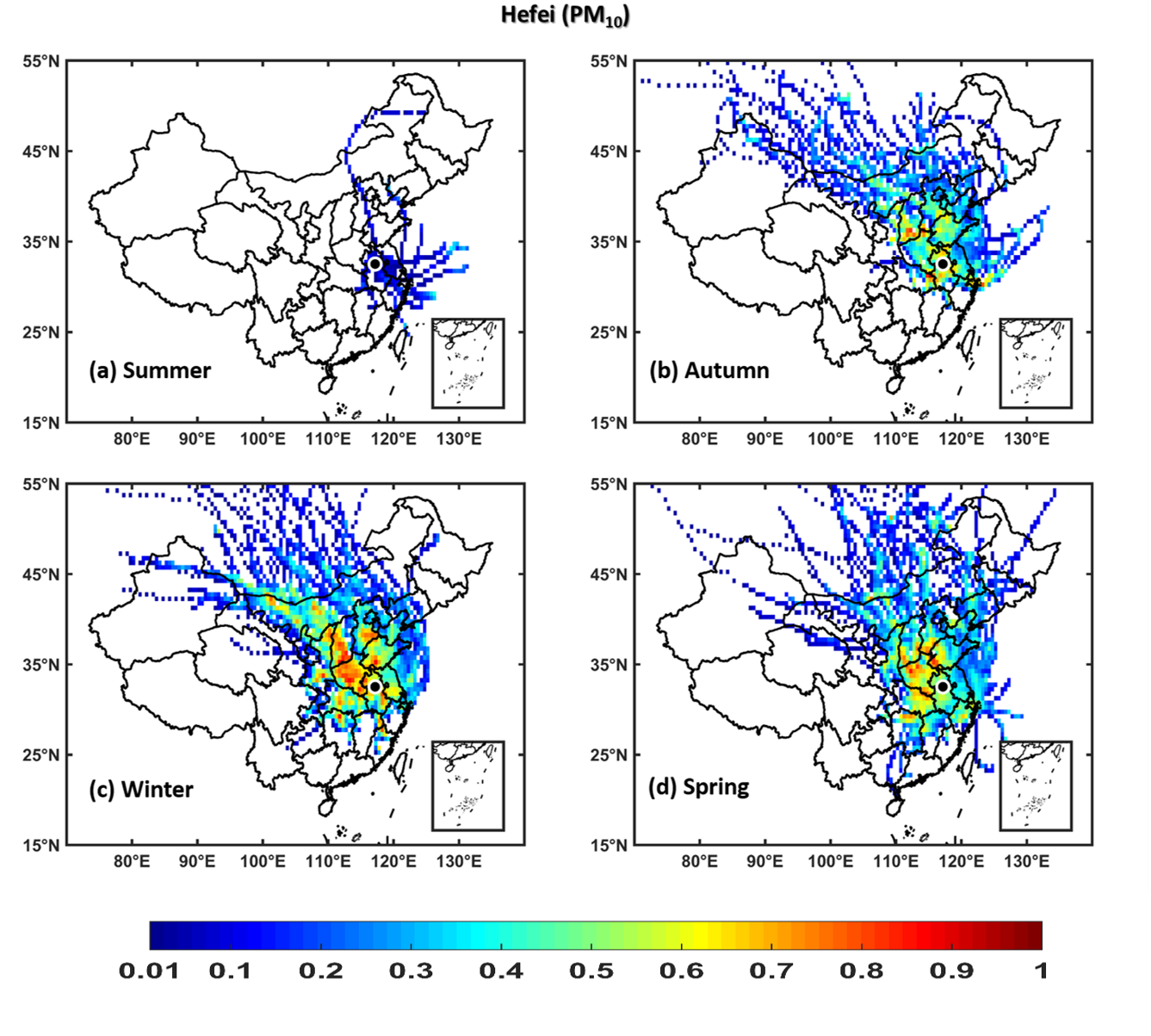


**Fig. S6.** PSCF Analysis based on PM_10_ grouped by season over Hefei. The color bar indicates the weights of Pollution source regions.


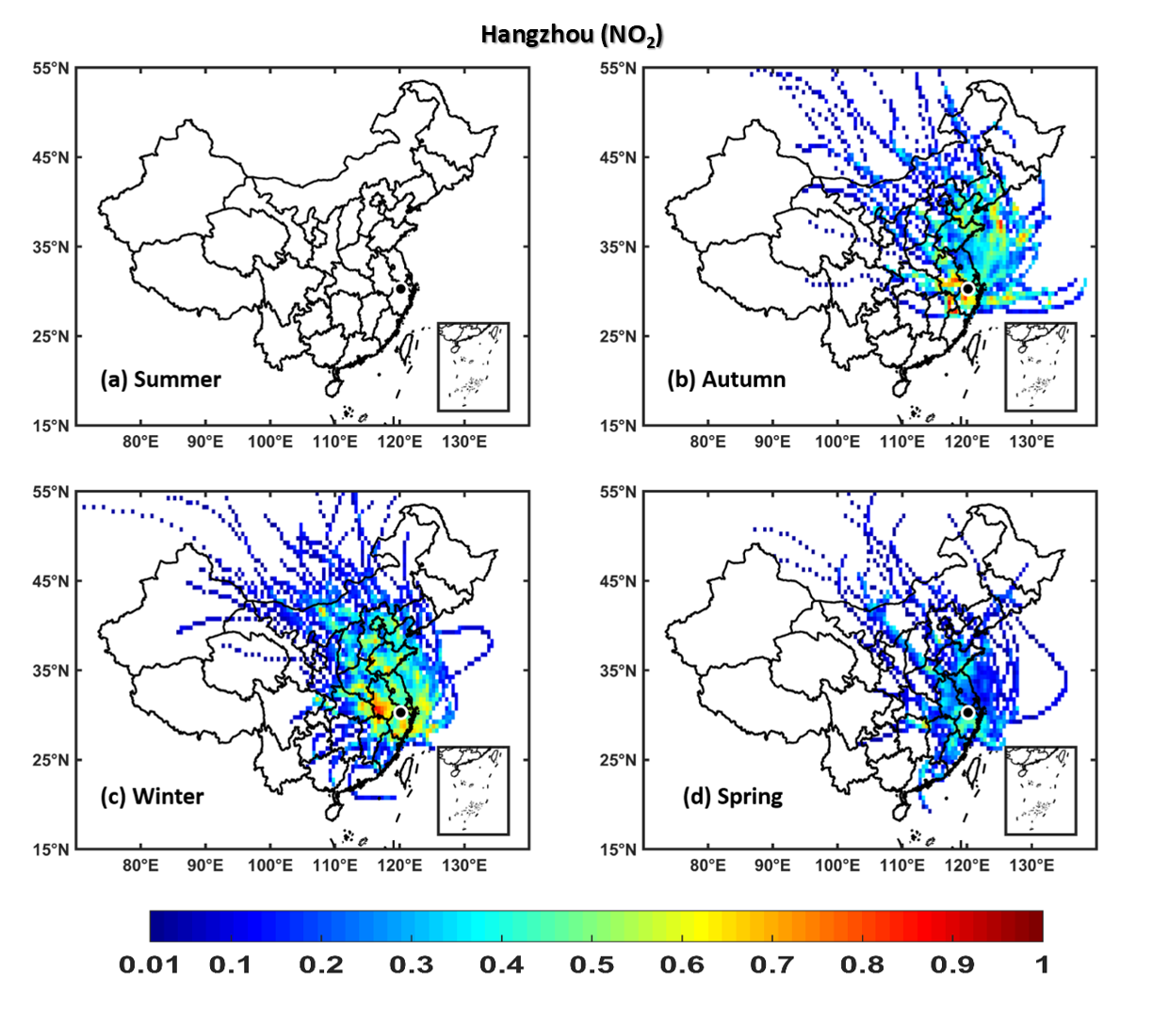


**Fig. S7.** PSCF Analysis based on NO_2_ grouped by season over Hangzhou. The color bar indicates the weights of Pollution source regions.


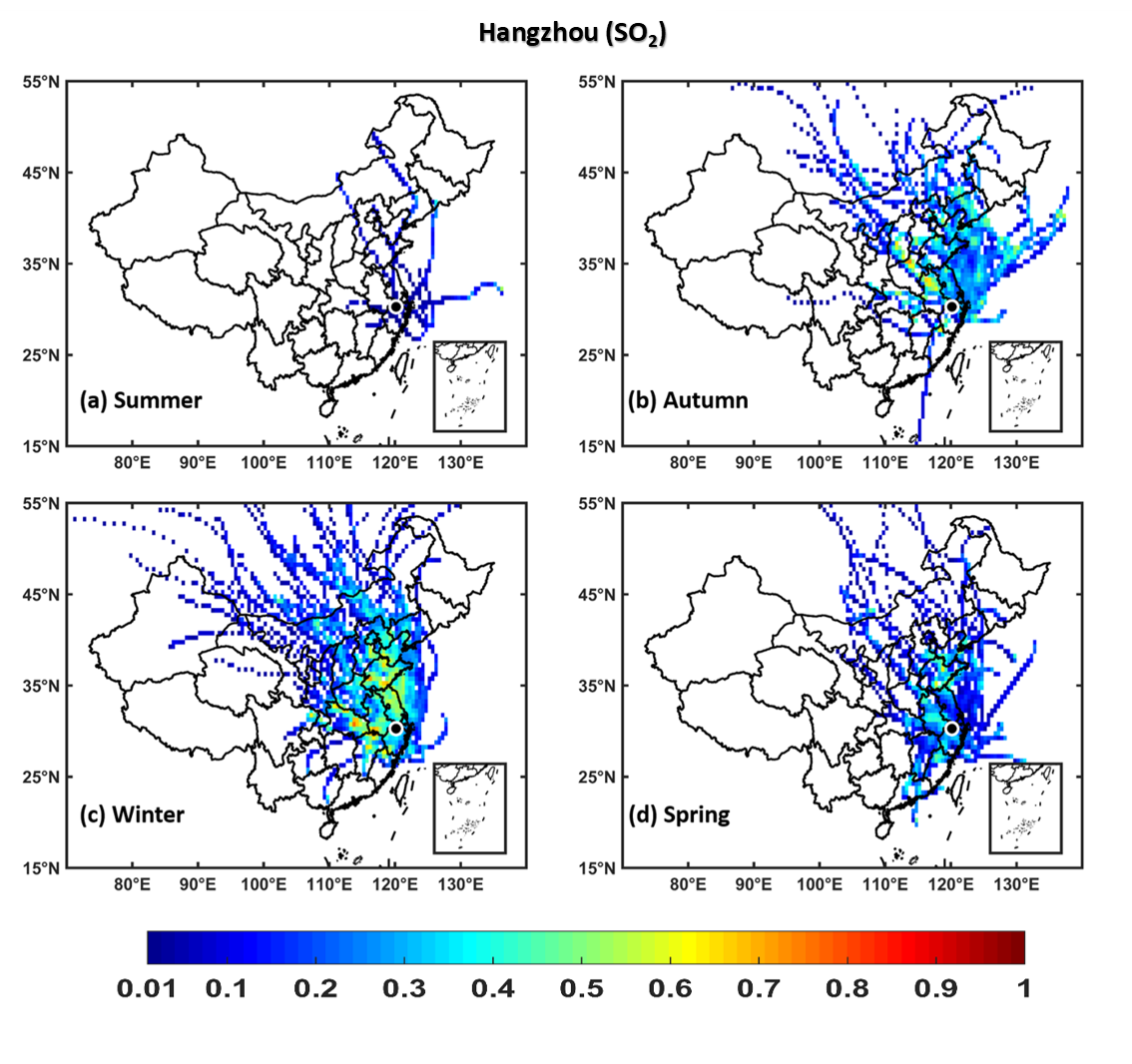


**Fig. S8.** PSCF Analysis based on SO_2_ grouped by season over Hangzhou. The color bar indicates the weights of Pollution source regions.


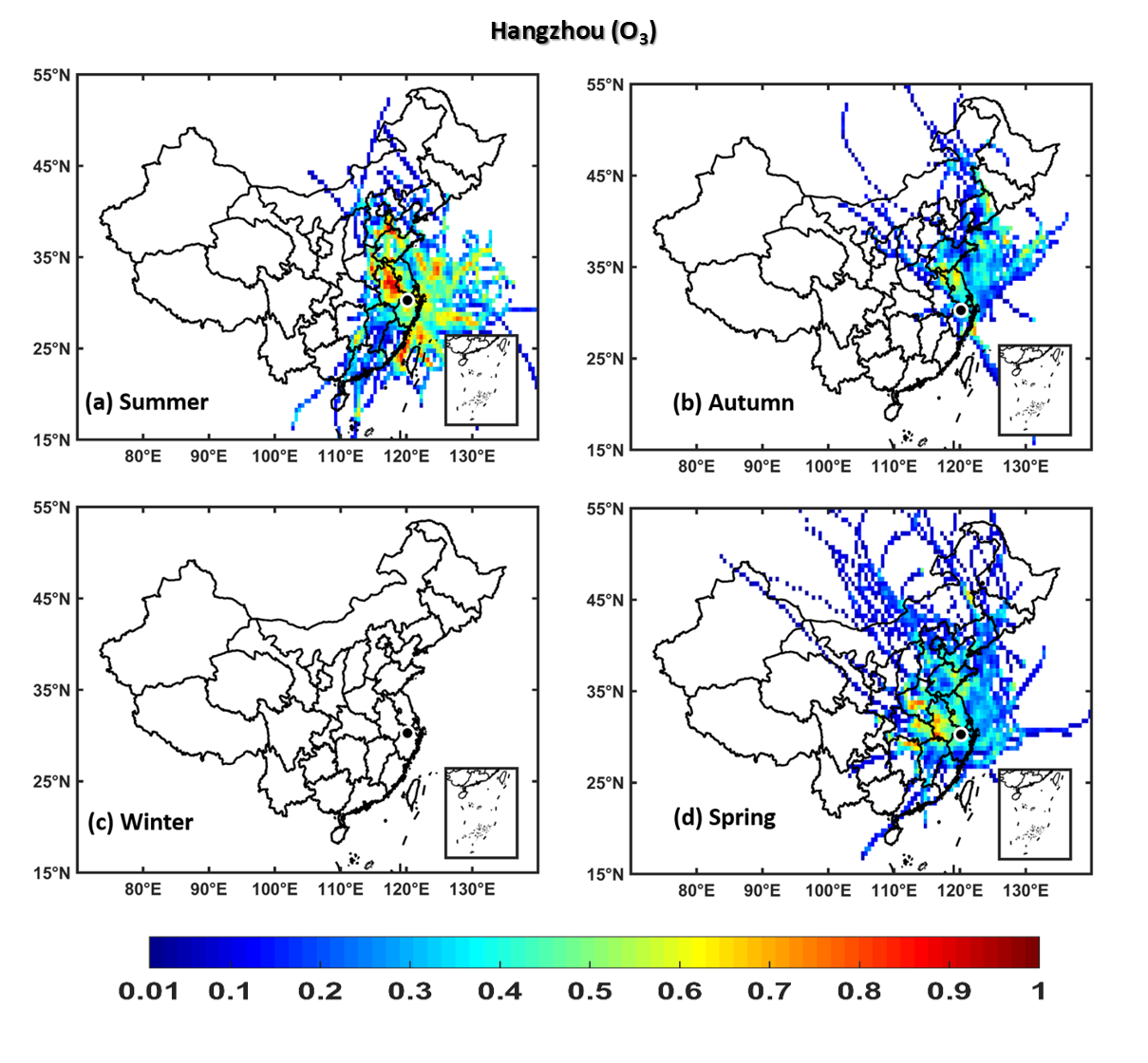


**Fig. S9.** PSCF Analysis based on O_3_ grouped by season over Hangzhou. The color bar indicates the weights of Pollution source regions.


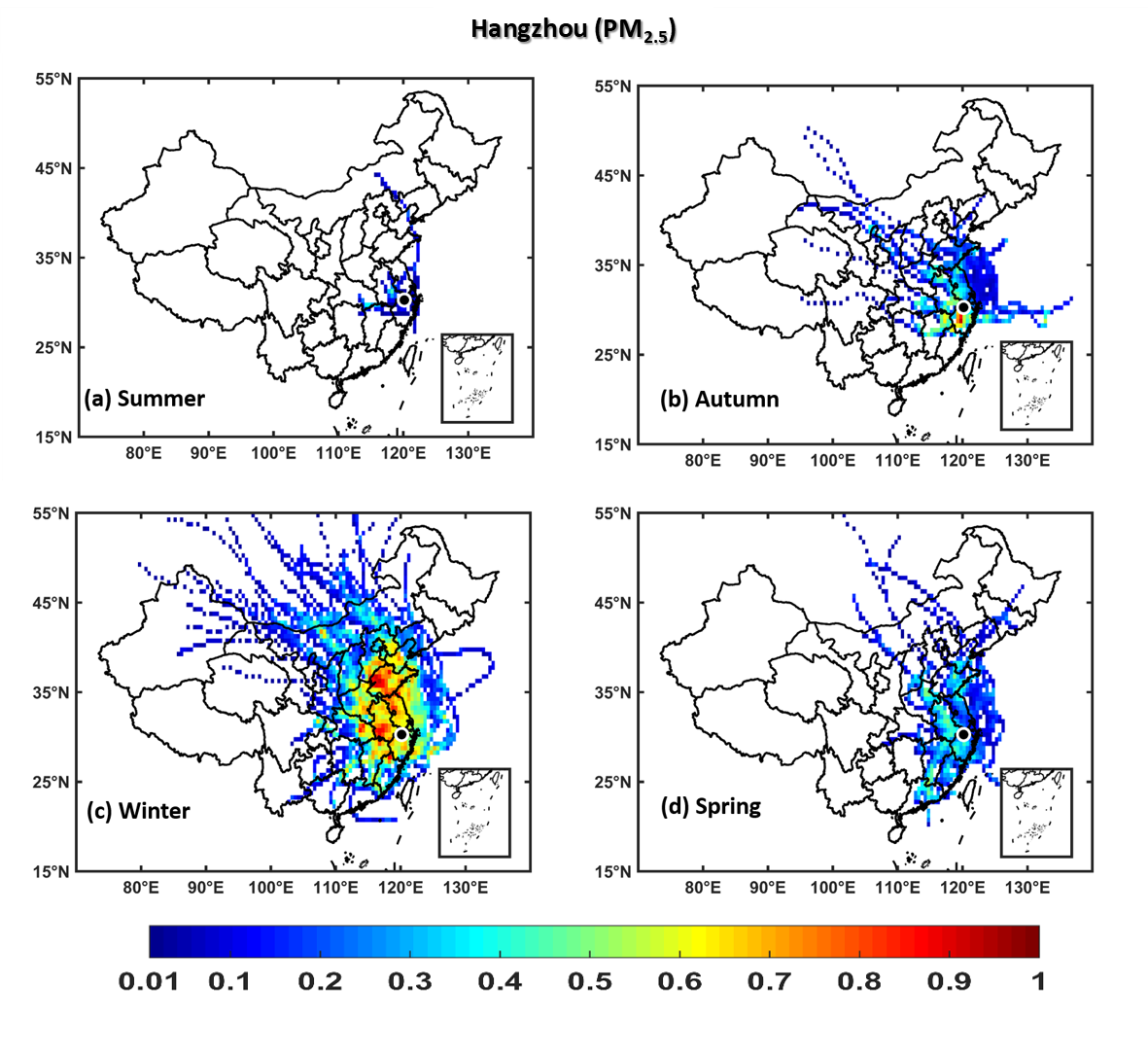


**Fig. S10.** PSCF Analysis based on PM_2.5_ grouped by season over Hangzhou. The color bar indicates the weights of Pollution source regions.


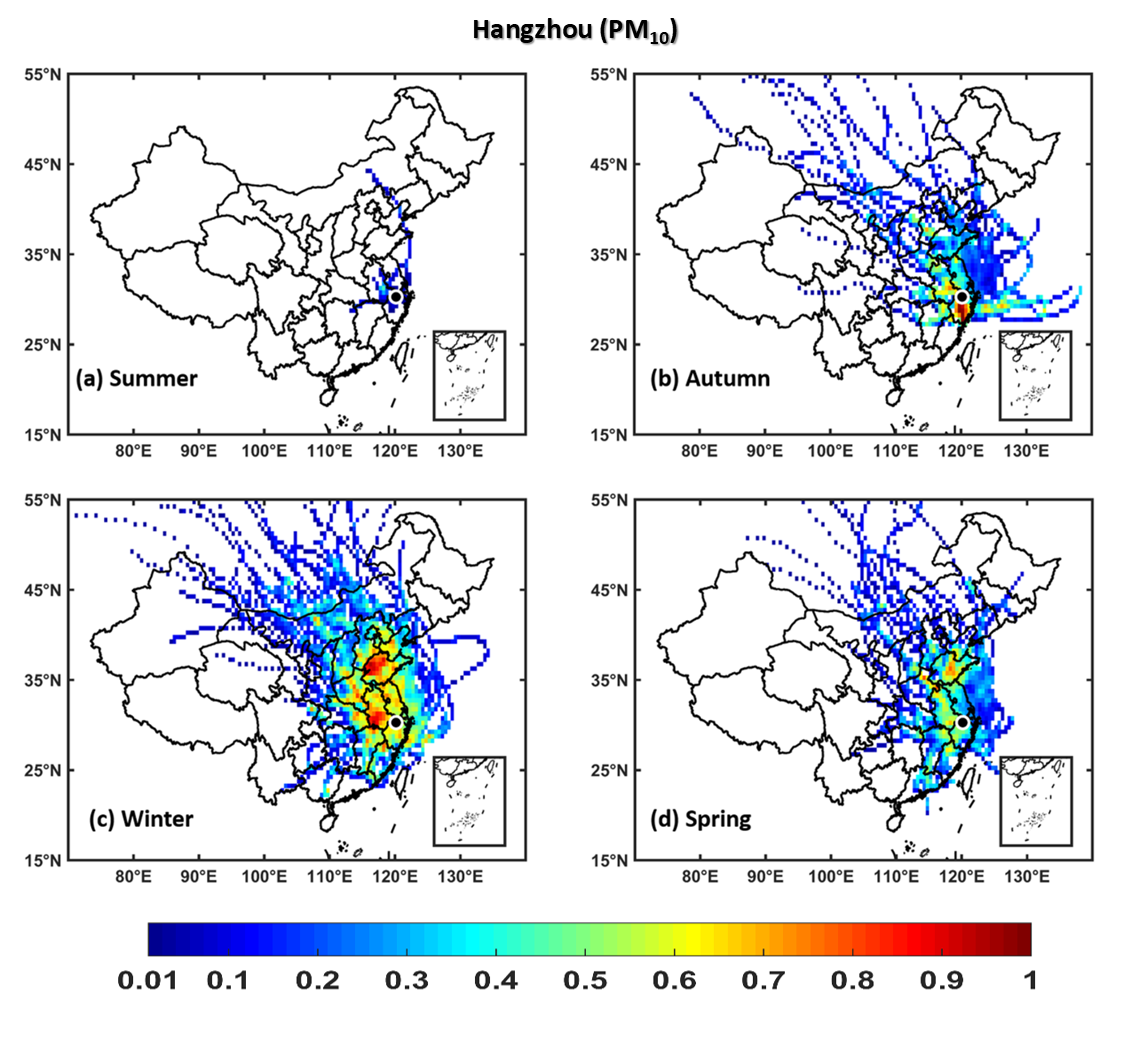


**Fig. S11.** PSCF Analysis based on PM_10_ grouped by season over Hangzhou. The color bar indicates the weights of Pollution source regions.


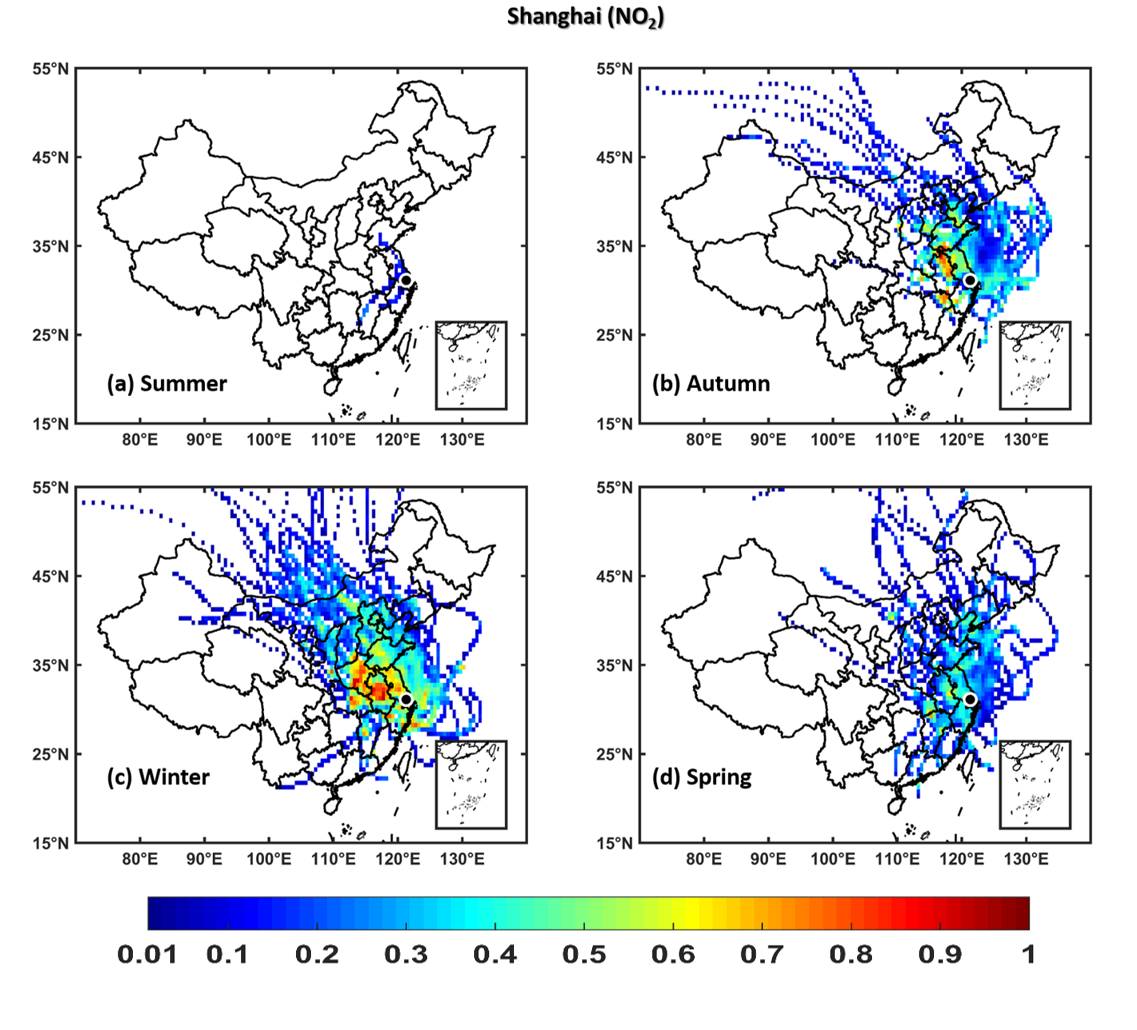


**Fig. S12.** PSCF Analysis based on NO_2_ grouped by season over Shanghai. The color bar indicates the weights of Pollution source regions.


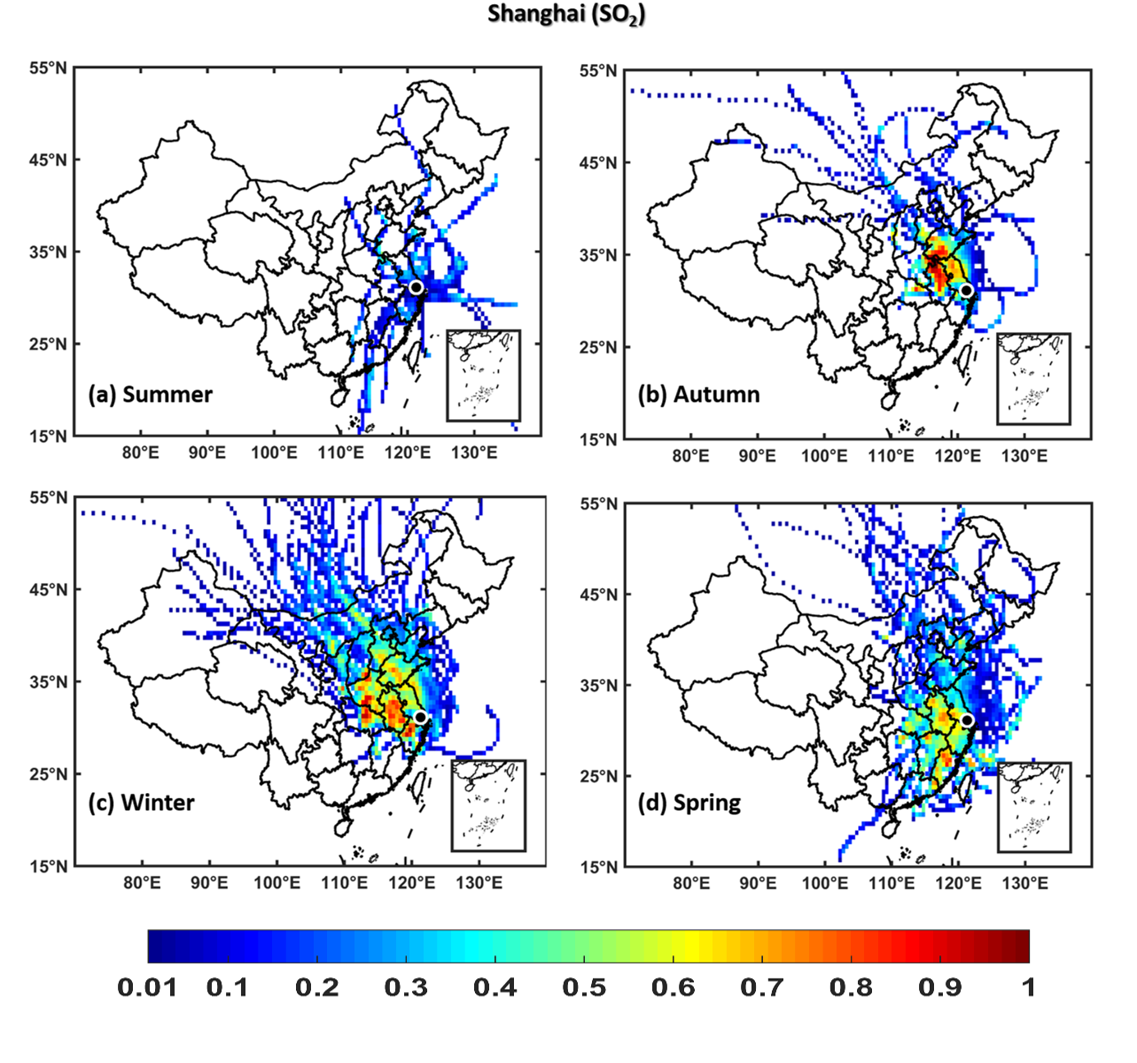


**Fig. S13.** PSCF Analysis based on SO_2_ grouped by season over Shanghai. The color bar indicates the weights of Pollution source regions.


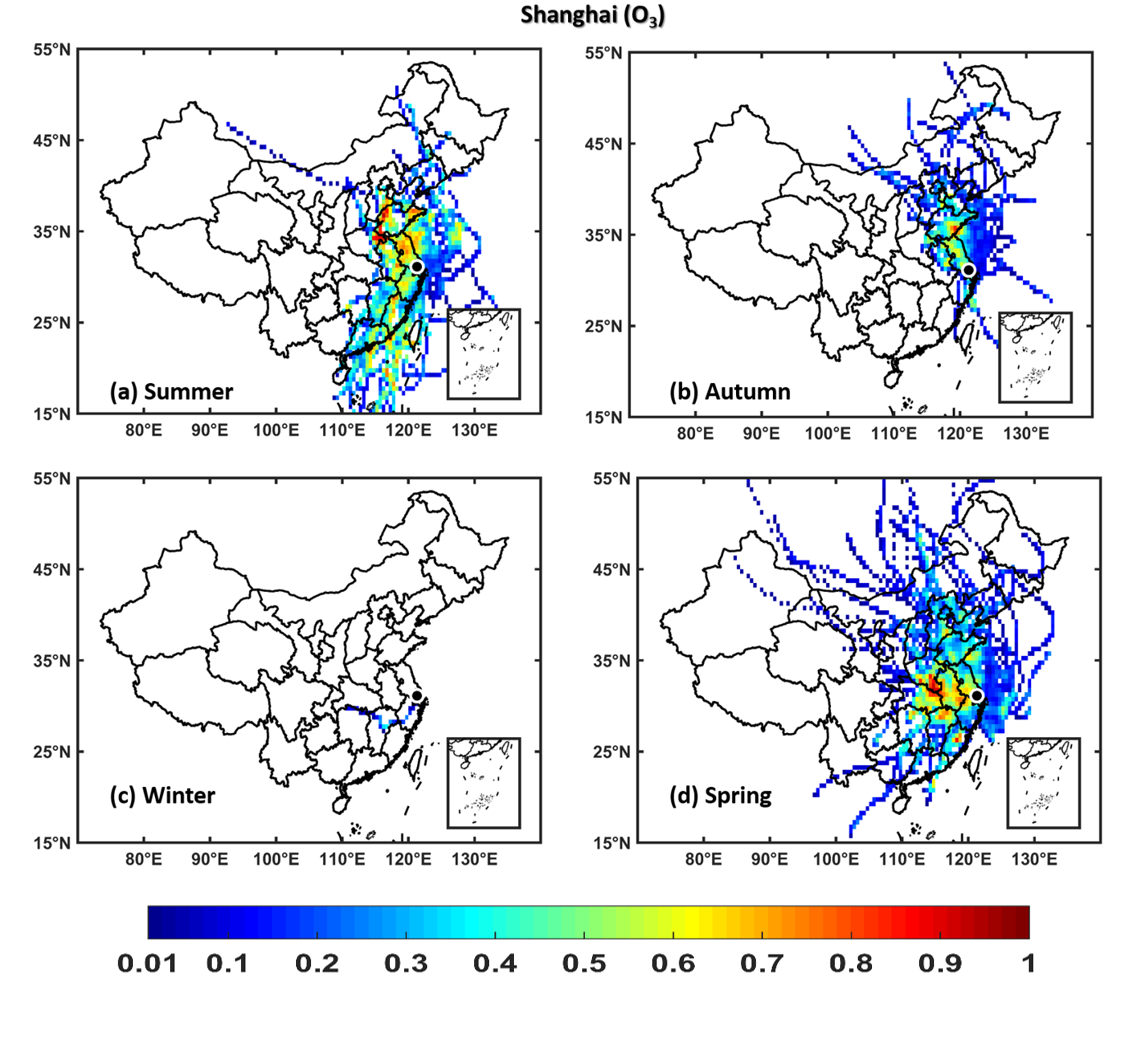


**Fig. S14.** PSCF Analysis based on O_3_ grouped by season over Shanghai. The color bar indicates the weights of Pollution source regions.


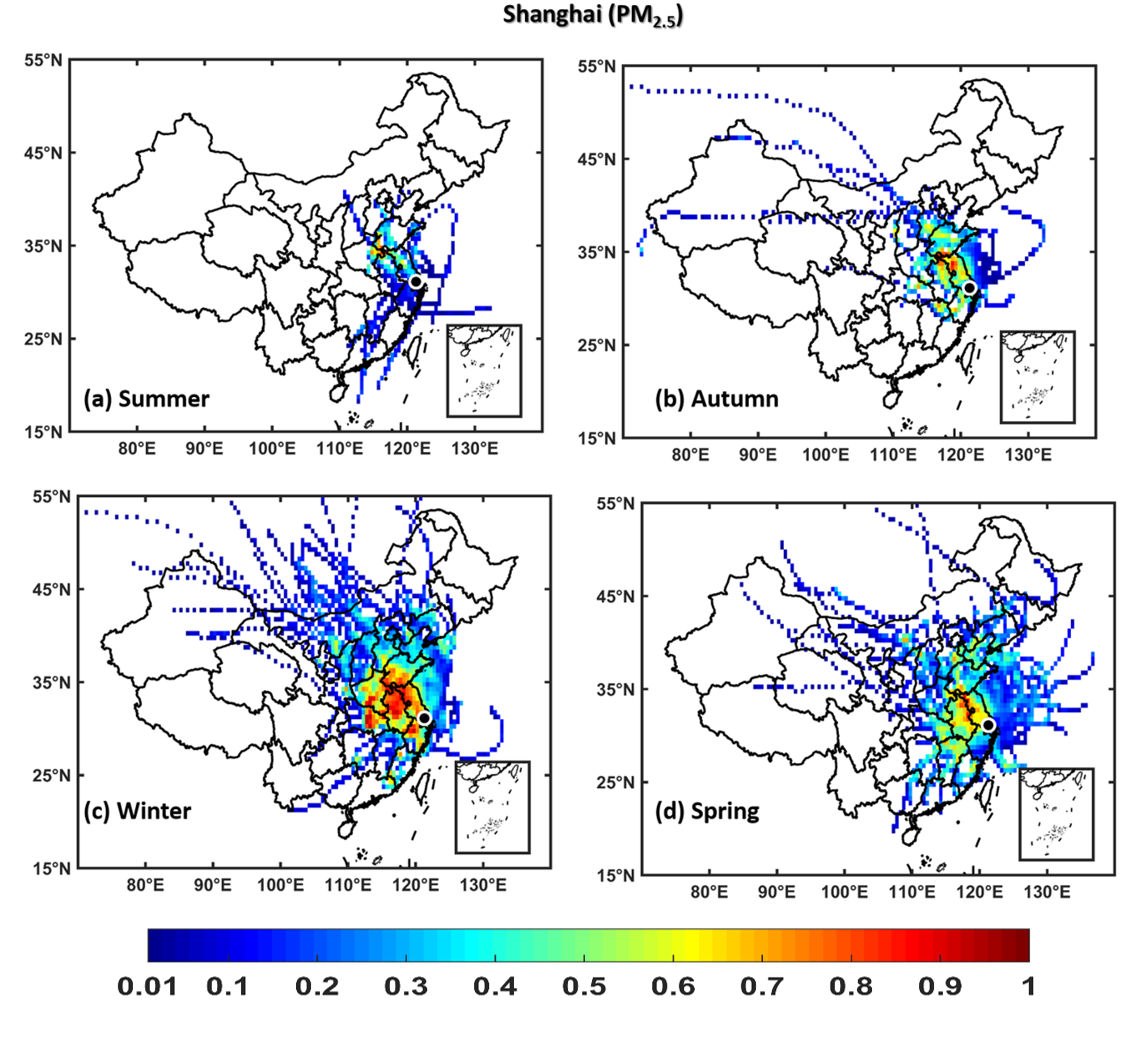


**Fig. S15.** PSCF Analysis based on PM_2.5_ grouped by season over Shanghai. The color bar indicates the weights of Pollution source regions.


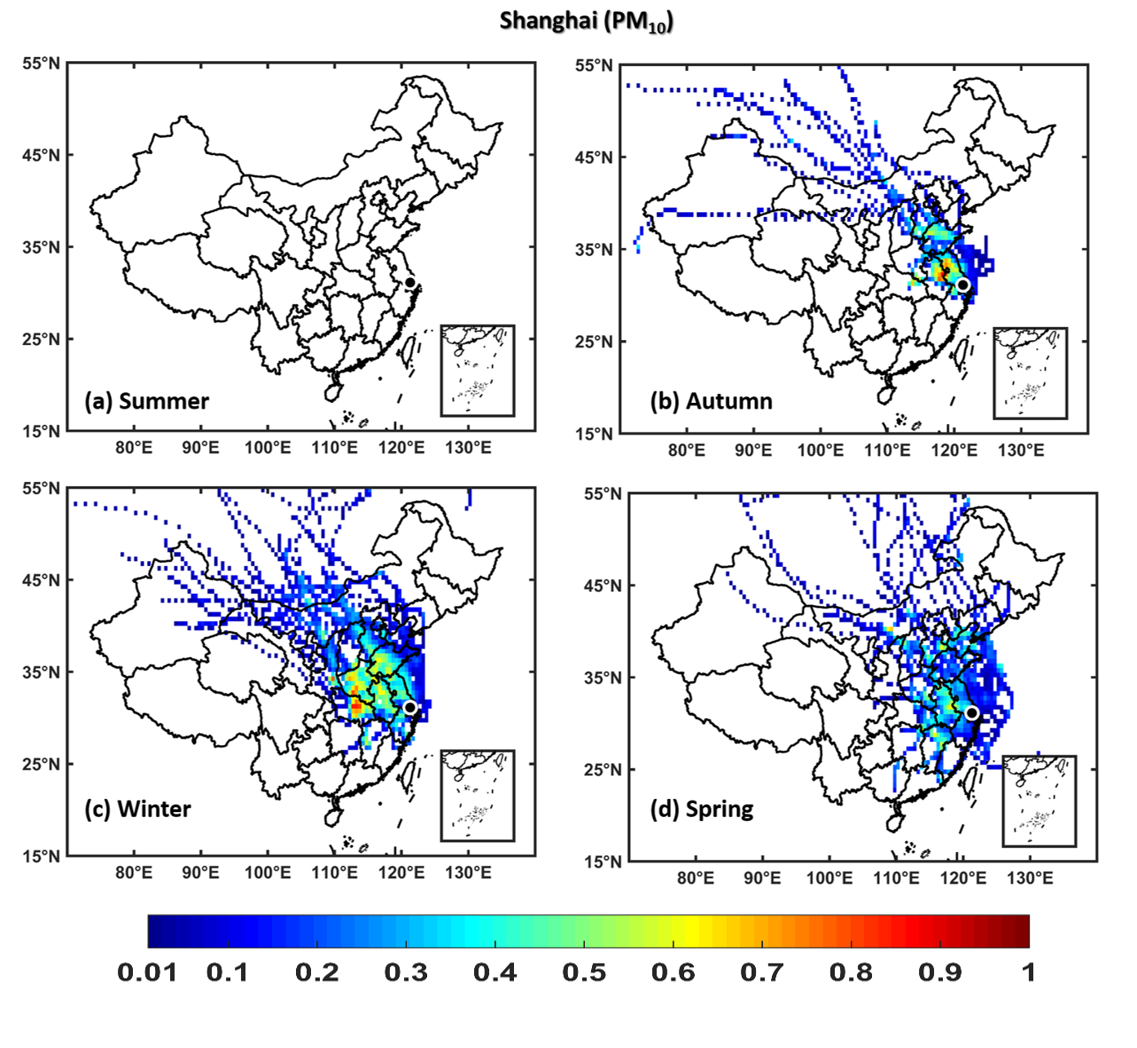


**Fig. S16.** PSCF Analysis based on PM_10_ grouped by season over Shanghai. The color bar indicates the weights of Pollution source regions.


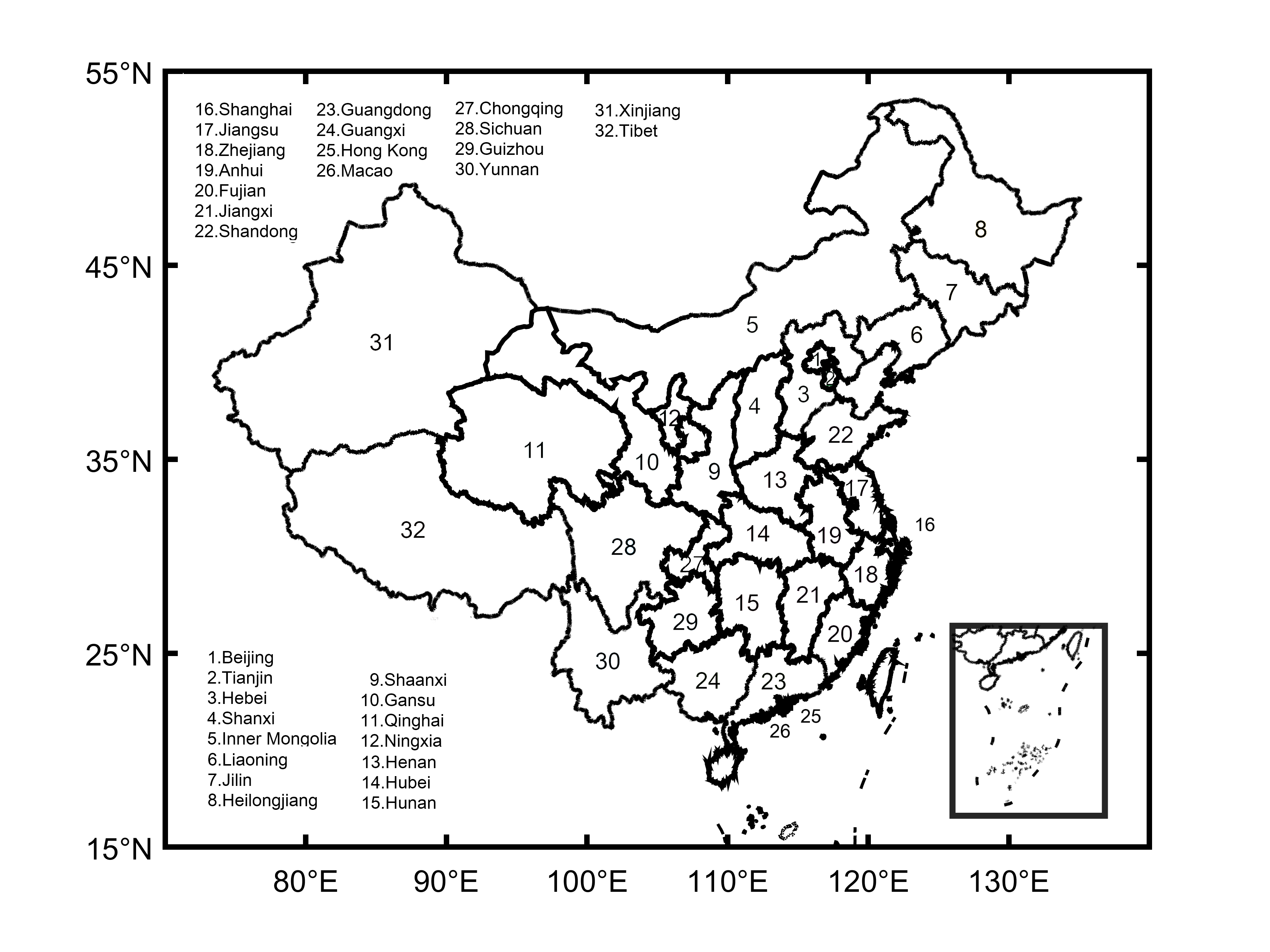


**Fig. S17.** Different regions of Peoples republic of China.
